# Integrin αVβ3 silencing sensitizes malignant glioma cells to temozolomide by suppression of homologous recombination repair

**DOI:** 10.18632/oncotarget.10897

**Published:** 2016-07-28

**Authors:** Markus Christmann, Kathrin Diesler, Dragomira Majhen, Christian Steigerwald, Nancy Berte, Halima Freund, Nikolina Stojanović, Bernd Kaina, Maja Osmak, Andreja Ambriović-Ristov, Maja T. Tomicic

**Affiliations:** ^1^ Department of Toxicology, University Medical Center Mainz, D-55131 Mainz, Germany; ^2^ Laboratory for Cell Biology and Signaling, Division of Molecular Biology, Ruđer Bošković Institute, HR-10000 Zagreb, Croatia

**Keywords:** malignant gliomas, integrin αVβ3, silencing, temozolomide, homologous recombination repair, Rad51

## Abstract

Integrins have been suggested as possible targets in anticancer therapy. Here we show that knockdown of integrins αVβ3, αVβ5, α3β1 and α4β1 and pharmacological inhibition using a cyclo-RGD integrin αVβ3/αVβ5 antagonist sensitized multiple high-grade glioma cell lines to temozolomide (TMZ)-induced cytotoxicity. The greatest effect was observed in LN229 cells upon integrin β3 silencing, which led to inhibition of the FAK/Src/Akt/NFκB signaling pathway and increased formation of γH2AX foci. The integrin β3 knockdown led to the proteasomal degradation of Rad51, reduction of Rad51 foci and reduced repair of TMZ-induced DNA double-strand breaks by impairing homologous recombination efficiency. The down-regulation of β3 in Rad51 knockdown (LN229-Rad51kd) cells neither further sensitized them to TMZ nor increased the number of γH2AX foci, confirming causality between β3 silencing and Rad51 reduction. RIP1 was found cleaved and IκBα significantly less degraded in β3-silenced/TMZ-exposed cells, indicating inactivation of NFκB signaling. The anti-apoptotic proteins Bcl-xL, survivin and XIAP were proteasomally degraded and caspase-3/−2 cleaved. Increased H2AX phosphorylation, caspase-3 cleavage, reduced Rad51 and RIP1 expression, as well as sustained IκBα expression were also observed in mouse glioma xenografts treated with the cyclo-RGD inhibitor and TMZ, confirming the molecular mechanism *in vivo*. Our data indicates that β3 silencing in glioma cells represents a promising strategy to sensitize high-grade gliomas to TMZ therapy.

## INTRODUCTION

Despite constant progress in medical care, high-grade gliomas (WHO grade III – anaplastic astrocytomas and grade IV–glioblastomas) remain incurable. Ionizing radiation (IR) combined with the methylating anticancer drug temozolomide (TMZ) represents the standard therapy of these tumors [1]. TMZ induces various DNA adduct*s* including *O*^6^-methylguanine (O^6^MeG), which is responsible for the anticancer activity. O^6^MeG can be repaired by the DNA repair protein O^6^-methylguanine-DNA methyltransferase (MGMT). In the absence of MGMT, O^6^-MeG leads, *via* replication and the involvement of mismatch repair, to DNA double-strand breaks (DSBs). Importantly, ~40% of all malignant gliomas are negative for MGMT [2, 3] and TMZ therapy is particularly effective in these tumors [4, 5]. However, ~60% of patients, whose tumors are proficient for MGMT, do not profit from the therapy. Thus, new strategies to overcome TMZ resistance in gliomas are urgently needed. One of these includes targeting of integrins.

Integrins are heterodimeric transmembrane glycoprotein α/β receptors that mediate cell adhesion and directly bind components of the extracellular matrix (ECM), thereby providing anchorage for cell motility and invasion. In addition, binding of integrins with ECM ligands induces a variety of intracellular signals and regulates cellular responses including proliferation, survival, migration and differentiation [6]. Activation of integrin receptors results in the association of multiple protein complexes, allowing integrins to transmit biochemical signals *via* tyrosine kinases such as focal adhesion kinase (FAK) or Src [7]. Integrin-associated proteins are involved in all major signal transduction pathways critical in determining the cell response to cytotoxic agents.

Integrins αVβ3 and αVβ5 are broadly expressed not only on blood vessels in brain tumors (glioblastomas), but also in tumor cells [8, 9]. Various pharmacological approaches for modulation of integrin signaling have been explored including antibodies and peptide-based agents [6, 10]. Indeed, treatment of tumors by integrin antagonist cilengitide (CGT) in the orthotopic brain model *in vivo* reduced tumor growth [11]. A clinical phase II study revealed that the concomitant and adjuvant addition of CGT, a cyclic αVβ3/αVβ5 RGD mimetic [12], to the standard TMZ radio-chemotherapy showed promising activity in glioblastoma patients with MGMT promoter methylation [13]. Unfortunately, in the phase III study (CENTRIC) CGT failed to show advantage in comparison to the standard treatment [14]. One reason out of many for this failure could be that mainly αVβ3 and αVβ5 expressed on endothelial cells were targeted, while integrins expressed on tumor cells were inefficiently blocked [15, 16]. Despite this inconclusive trial, integrins still remain an attractive target for cancer therapy, which is strongly supported by the present study. Also, the newest data conducted on tumor material of the CORE study (failed to show benefit of CGT in patients with MGMT positive tumors) [17] showed that αVβ3 expression correlates with better OS and PFS in CGT-treated patients with tumors expressing MGMT [18].

Since integrins promote many essential cellular functions, their knockdown by means of siRNA might be a promising approach to enhance the efficacy of tumor therapy. Here, we particularly focused on molecular pathways/signaling initially caused by silencing of integrin β3 in glioblastoma cells. We show in cell culture *in vitro* and in a xenograft model *in vivo* that β3 silencing suppresses DNA repair of TMZ-induced DSBs impairing homologous recombination (HR). Furthermore we provide evidence of the involvement of the Akt/NFκB signaling pathway in this process.

## RESULTS

### Determination of integrin status in human malignant glioma cell lines

Expression of integrin heterodimers (α3β1, α4β1, αVβ3 and αVβ5), together with MGMT and p53 status in a panel of ten cell lines is shown in Table 1 and Supplementary Figure S1 (histograms). The p53 and MGMT activity (Table 1) were determined before [2, 3, 19]. Only two of the glioma cell lines (GBP61 and U138MG) were shown to express all four integrin heterodimers. Nine out of ten cell lines express αVβ3, indicating that this integrin might be a suitable therapeutic target for malignant gliomas. A total of seven cell lines were shown to express the α4β1 integrin. Out of the nine cell lines expressing αVβ3, we selected four lines for further investigation. These cell lines do not show MGMT activity which enabled us to achieve maximum TMZ cytotoxicity without MGMT inhibitor. We chose the U138MG cell line, showing expression of all four integrins, the glioblastoma cell lines LN229 and LN308, expressing αVβ3 and α4β1, but are characterized by a different p53 status [19, 20] and the U87MG cell line expressing αVβ3 and αVβ5, also used in the xenograft model.

**Table 1:** Integrin expression in malignant glioma cell lines

Cell line	p53 status (rest activity)	MGMT activity (fmol/mg protein)	α3β1	α4β1	αvβ3	αvβ5
T98MG	mt (0%)	+ (428)	−	+	+	+
GBP61	wt	+ (293)	+	+	+	+
LN18	mt (1%)	+ (205)	−	+	+	+
LN229	wt	− (4)	−	+	+	−
LN308	mt (deletion)	− (1)	−	+	+	(+)
LN319	mt (1%)	− (0)	+	(+)	(+)	+
LN428	mt (0%)	− (0)	−	−	+	(+)
D247MG	wt	− (19)	−	+	−	−
U87MG	wt	− (1)	−	−	+	+
U138MG	mt (9%)	− (2)	+	+	+	+

wt, wild-type; mt, mutant; “+“, relative strong expression (MFI = 0.4–1.0); “(+)“, relative weak expression (MFI = 0.1–0.4); “–“, no expression (MFI = 0). Mean fluorescence intensity (MFI) was set to 1 relative to the positive control (αVβ3 signal in GBP61 cells).

### Integrin β3, β5, αV, α3 and α4 silencing sensitizes malignant glioma cells to TMZ

**Figure 1:**
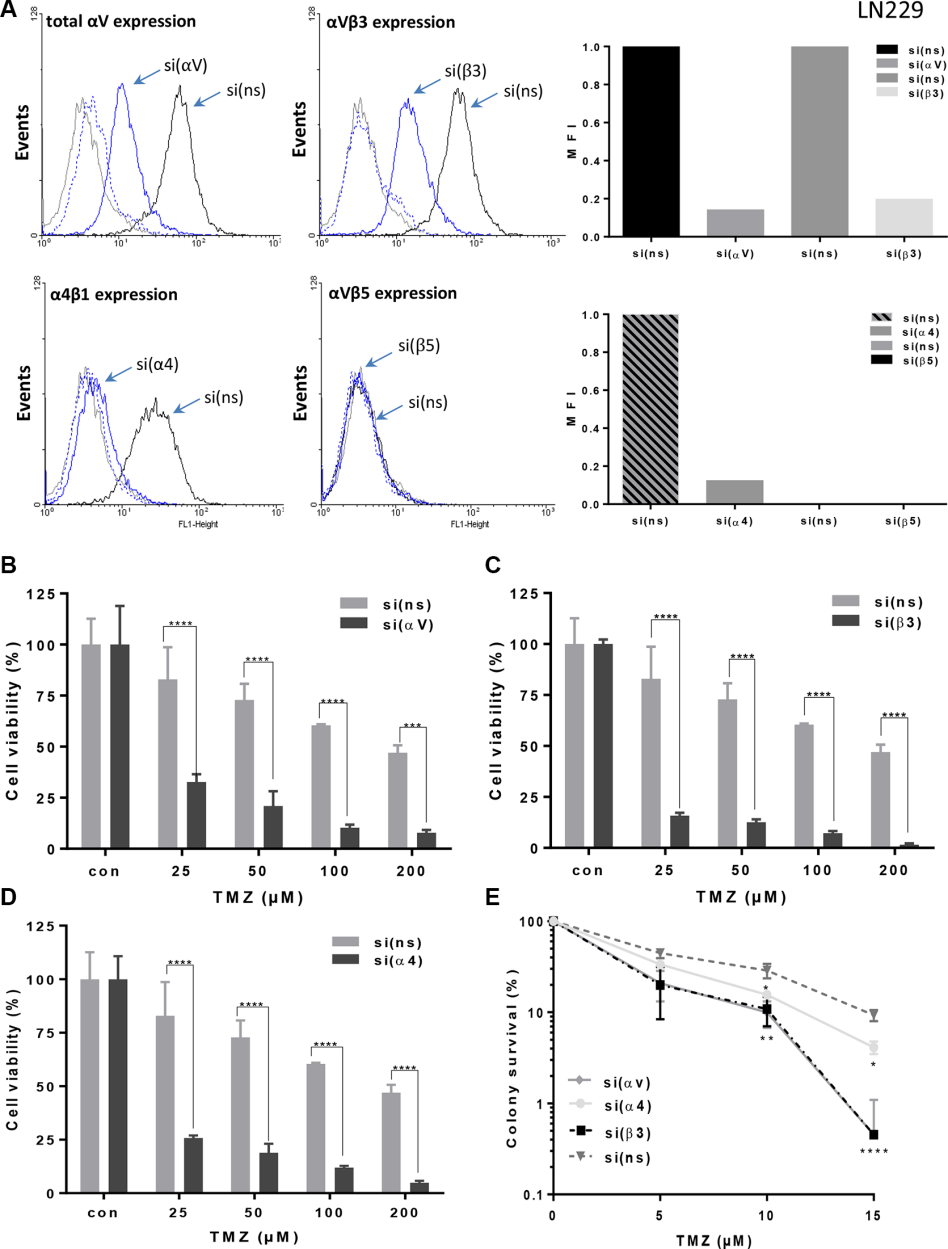
Integrin expression and sensitization to TMZ after specific integrin silencing (**A**) LN229 cells were transfected with integrin αV-, α4-, β3-, β5-or control non-silencing (ns)-siRNA, 48 h later the cells were detached by EDTA and the expression of αVβ3, αVβ5, αV, and α4β1 was measured by flow cytometry. As a negative control, mouse IgG antibodies were used. The expression i.e. effective integrin silencing was quantified by mean fluorescence intensity (MFI) and represented as relative to expression in cells transfected with control non-silencing (ns)-siRNA that was set to 1. The data presented are representative of three independent experiments with similar results. (**B**–**D**) LN229 cells were transfected with integrin αV-, α4-, β3-, or ns-siRNA, and 24 h later collected by trypsinization and seeded for cell viability assay (MTT). After another 24 h the cells were treated with TMZ and after six days metabolic activity was assessed. The data are the mean of three independent experiments in quadruplicates ± SD. (**E**) LN229 cells were seeded in 6-well cluster and 24 h later transfected with integrin αV-, α4-, β3- or ns-siRNA. After additional 24 h cells were re-seeded for colony formation assay at the density of 1000 cells per 6-well dish, and 24 h later exposed to TMZ or left unexposed. Fourteen days later the visible colonies were fixed, stained and counted. The plating efficiency of the cell lines after integrin silencing was ~60–70%. Unexposed integrin-specific and ns-control were set to 100%. The data are the mean of three independent experiments ± SD. *p ≤ 0.05 significant, **p ≤ 0.01 very significant, ***p ≤ 0.005 highly significant, ****p ≤ 0.001 most significant.

In all analyzed high-grade glioma cell lines (LN229, LN308, U138MG, U87MG) the integrin subunit-specific siRNA transfection led to reduction in expression of corresponding integrins, whereas the control-siRNA had no effect. A representative example of integrin expression and of effective silencing, as quantified by mean fluorescence intensity (MFI), is shown for the LN229 cell line (Figure 1A). In order to concomitantly reduce the expression of αVβ3 and αVβ5, in some experiments cells were transfected with integrin αV-specific siRNA.

Silencing of the corresponding integrin subunits in the cell lines LN229, LN308, U87MG and U138MG clearly increased sensitivity to TMZ compared to the control-siRNA, as shown by the MTT assay (for LN229 cell line, see Figure 1B–1D, for other cell lines, see Supplementary Figure S2–S4). Of note, the cells were exposed to TMZ for six days. Growth rates of the cell lines upon siRNA transfection were slowed down by 20% at maximum relative to the non-transfected control. We also determined reproductive cell survival by colony formation assay (LN229 Figure 1E and U138MG Supplementary Figure S2B) showing that each of the integrin-specific siRNAs led to a reduced clonogenic survival as compared to control-siRNA. The silencing of the integrin subunits β3 led to the strongest sensitization effect upon TMZ exposure in all analyzed cell lines indicating that the integrin αVβ3 plays a crucial role in mediating TMZ sensitivity. The strongest sensitization effect was achieved in LN229 cells, thus we used this cell line for all further experiments.

### Integrin silencing and cyclo-RGD integrin inhibitor sensitize glioma cells to TMZ-induced apoptosis

**Figure 2:**
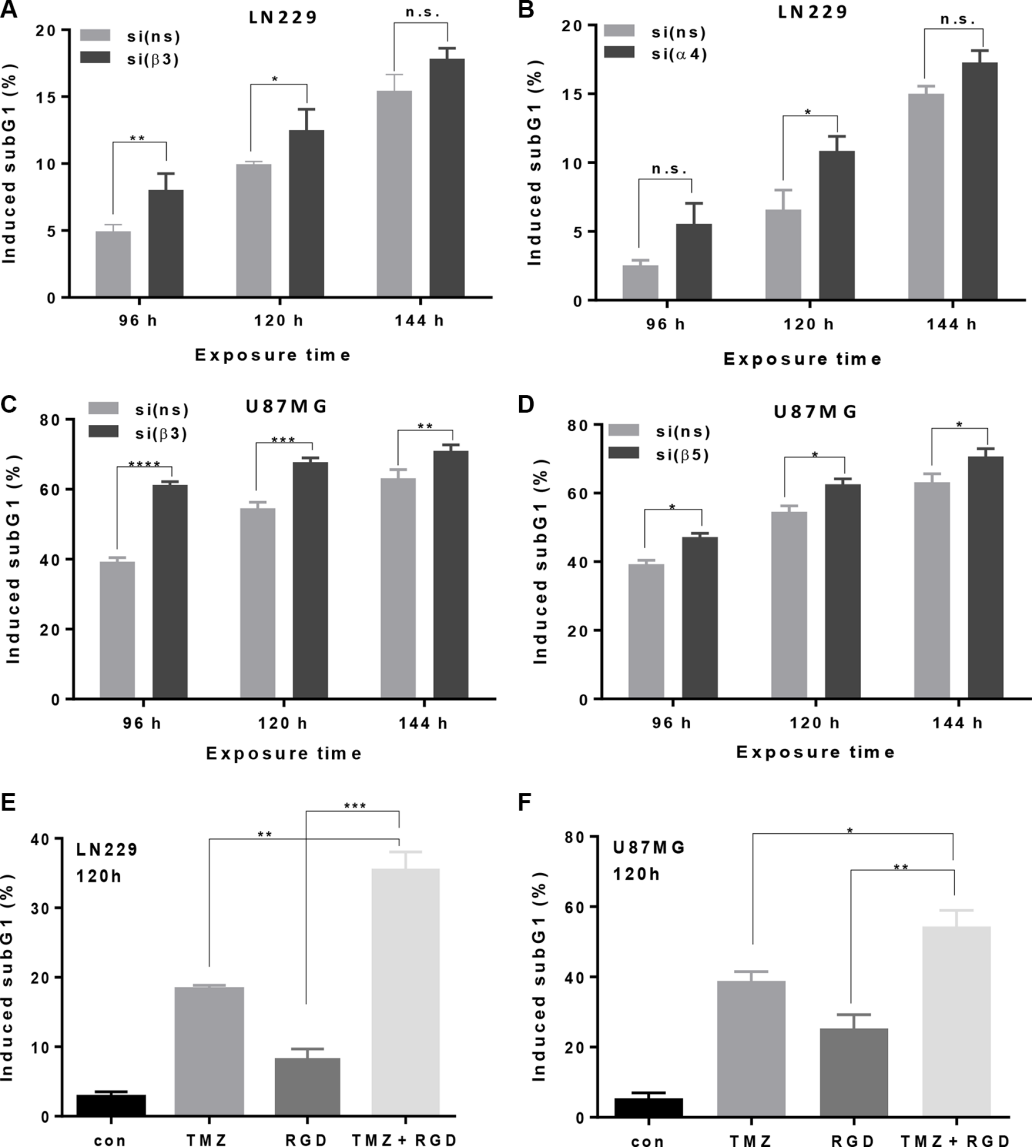
Modulation of apoptosis induction by integrin silencing or pharmacological inhibition (**A**, **B**) LN229 and (**C**, **D**) U87MG cells were transfected with integrin β3-, α4-, β5- or ns-siRNA, and 48 h later exposed to 100 μM TMZ. After 96, 120 and 144 h the cells were subjected to subG1 flow cytometric analysis. The data are the mean of two independent experiments in duplicates ± SD. (**E**, **F**) LN229 and U87MG cells were pre-treated with a cyclo-RGD integrin antagonist (10 μg/mL) alone, or exposed, in addition, to 100 μM TMZ. 120 h later, the cells were subjected to subG1 flow cytometric analysis. The data are the mean of two independent experiments in duplicates ± SD. *p ≤ 0.05 significant, **p ≤ 0.01 very significant, ***p ≤ 0.005 highly significant, ****p ≤ 0.001 most significant. n.s., not significant.

**Figure 3:**
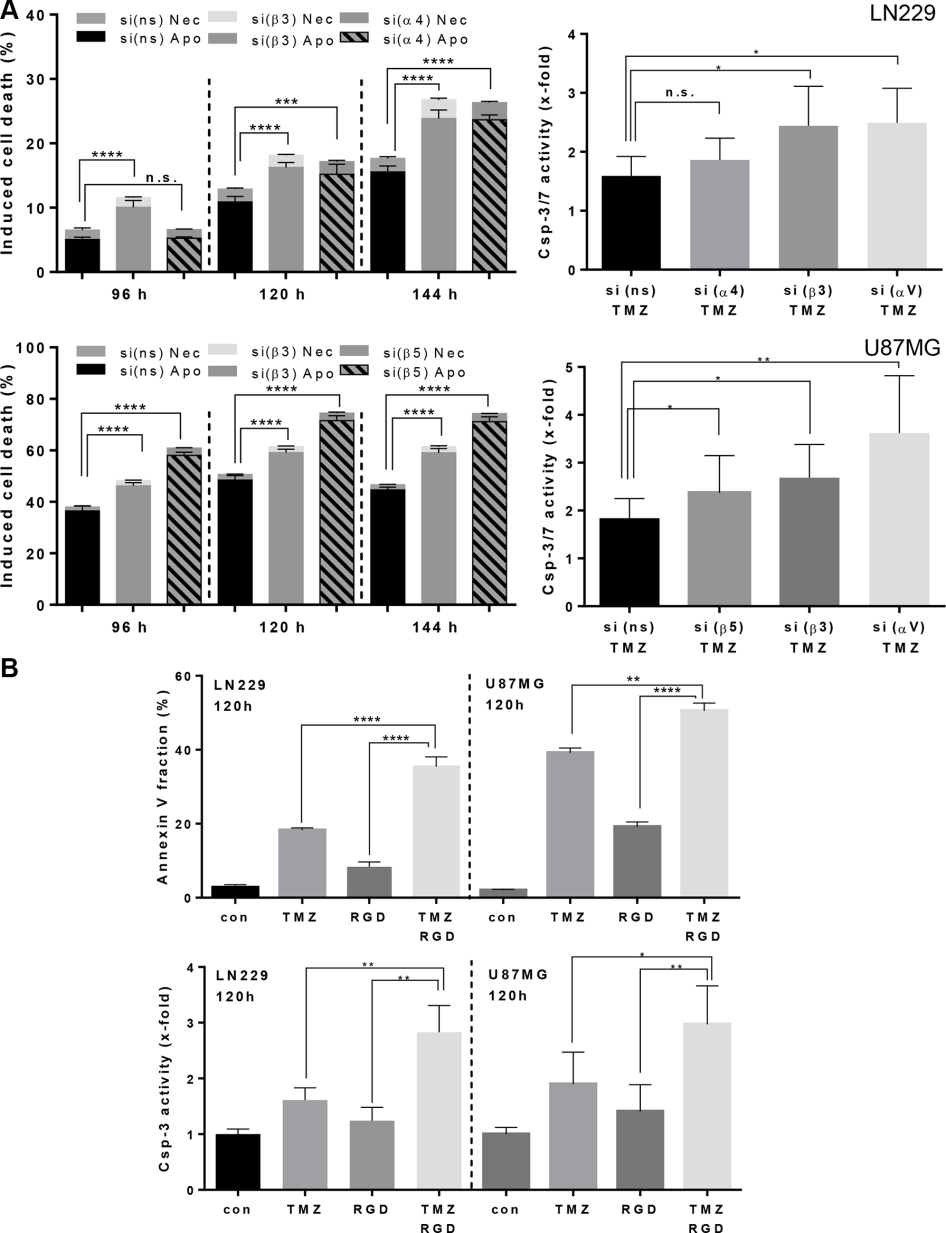
Induction of apoptosis/necrosis and caspase-3/7 activity (**A**) (left panel): LN229 and U87MG cells were transfected with integrin β3-, α4-, β5- or ns-siRNA, respectively, and 48 h later exposed to TMZ (100 μM). After 96, 120 and 144 h the cells were subjected to subG1 flow cytometric analysis. The data are the mean of two independent experiments in duplicates ± SD. (**A**) (right panel): LN229 and U87MG cells were transfected with integrin β3-, α4-, β5- or ns-siRNA, respectively, and 24 h later re-seeded in the 96-well plate. After additional 24 h cells were treated with TMZ (100 μM) and 120 h later the caspase-3/7 activity was determined. (**B**) LN229 and U87MG cells were pre-treated with a cyclo-RGD integrin antagonist (10 μg/mL) alone, or exposed, in addition, to TMZ (100 μM). 120 h later, the cells were subjected to annexin V/PI staining and to caspase-3/7 analysis. The data are the mean of two independent experiments in duplicates ± SD. *p ≤ 0.05 significant, **p ≤ 0.01 very significant, ***p ≤ 0.005 highly significant, ****p ≤ 0.001 most significant. n.s., not significant.

TMZ predominantly induces apoptosis in malignant glioma cells as a very late event [21]. A concentration of 100 μM TMZ was shown to be an optimal concentration in glioma cell culture experiments [21, 22] and is also achievable in the blood plasma. Integrin silencing sensitized cells to TMZ, as shown by the subG1 fraction, determined at 96, 120 and 144 h (for LN229 and U87MG, see Figure 2A–2D; for LN308 and U138MG, see Supplementary Figure S5A and S6A). The same was true using the annexin V assay, pointing to a specific induction of apoptosis, whereas the frequency of necrosis remained constant (for LN229 and U87MG, see Figure 3A, left panel). In support of this, increased activity of the executive caspase-3/7 was observed (for LN229 and U87MG, see Figure 3A, right panel; for LN308 and U138MG, see Supplementary Figure S7A, S7B, left panel). Of note, integrin silencing slowed down the cells in general but did not cause any significant changes in the cell cycle phase distribution (data not shown).

In order to elucidate whether the sensitization effect towards TMZ can also be achieved by administration of a cyclo-RGD inhibitor, we used an integrin antagonist that is similar to CGT. This inhibitor predominantly inhibits integrin αVβ3 and, albeit to a lower extent, αVβ5, thus the imposed effects can be primarily attributed to inhibition of αVβ3. As shown by the increased subG1 fractions all glioma cell lines were sensitized to TMZ, independent of their p53 status (Figure 2E, Figure 2F, Supplementary Figure S5B and Supplementary Figure S6B). The strongest inhibitor-mediated sensitization effect, in analogy to integrin β3 and αV knockdown, was achieved in LN229 cells (Figure 2E). The induction of apoptosis upon combination treatment with the cyclo-RGD inhibitor and TMZ was confirmed by the annexin V assay (Figure 3B, upper panel) and induction of the caspase-3/7 activity (Figure 3B, lower panel, Supplementary Figure S7A, S7B, right panel).

### Effect of αVβ3 silencing on the number of γH2AX foci upon TMZ exposure

**Figure 4:**
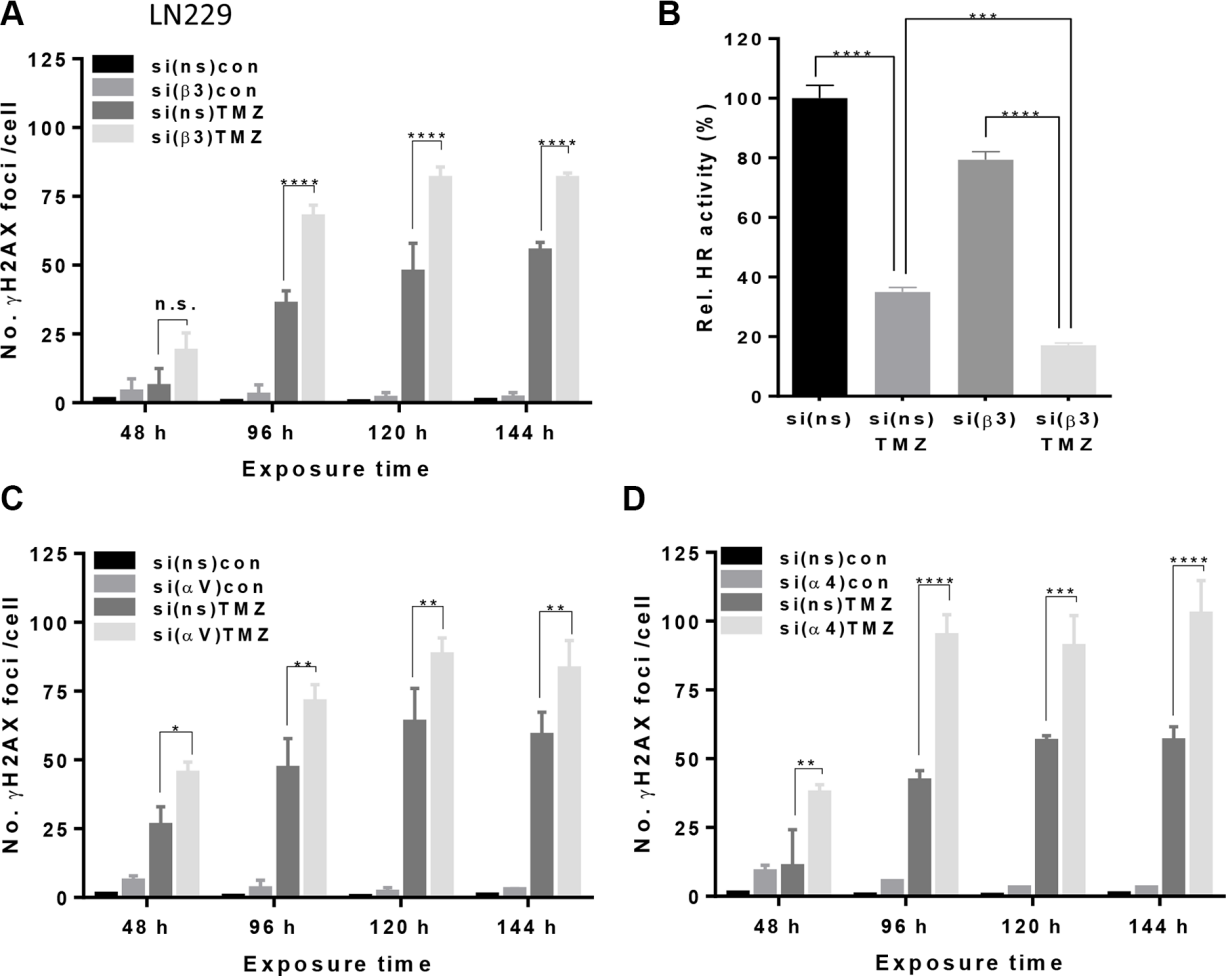
Induction of γH2AX foci upon TMZ/integrin silencing (**A**, **C**, **D**) LN229 cells were transfected with β3-, αV-, α4- or ns-siRNA and exposed to 100 μM TMZ. At indicated time points, the cells were stained for γH2AX. Counterstaining of the nuclei was performed using TO-PRO-3. The data are the mean of two independent experiments in duplicates ± SD. (**B**) Efficiency of homologous recombination (HR) after transfection of LN229 cells with control-siRNA or β3-siRNA alone or combined with 100 μM TMZ. Cellular DNA was extracted 96 h after TMZ exposure. Samples were standardized with universal primers, detecting the plasmid backbone for control of transfection efficiency. HR activity in control-siRNA transfected cells was set to 100%. The data are the mean of two independent experiments in duplicates ± SD. *p ≤ 0.05 significant, **p ≤ 0.01 very significant, ***p ≤ 0.005 highly significant, ****p ≤ 0.001 most significant. n.s., not significant.

To investigate whether integrin signaling is associated with the DNA damage response and DNA repair, we determined the induction of γH2AX foci in integrin-silenced cells exposed to TMZ. We observed that the number of TMZ-induced γH2AX foci was enhanced by all integrin-specific siRNAs in LN229 cells (Figure 4A–4D) and U138MG cells (Supplementary Figure S8), indicating inefficient repair of DSBs. In unexposed controls, none of the siRNAs was able to induce γH2AX foci on its own. The repair foci were verified by co-localization of γH2AX and 53BP1 (data not shown). Since the sensitization to TMZ using either β3- or αV-siRNA in LN229 cells was comparable (see Figure 1B, 1C and 1E), the increased number of γH2AX foci is likely caused by silencing of αVβ3 but the effect of αVβ1 cannot be excluded.

### Effect of αVβ3 silencing on the number of Rad51 foci and HR efficiency upon TMZ exposure

**Figure 5:**
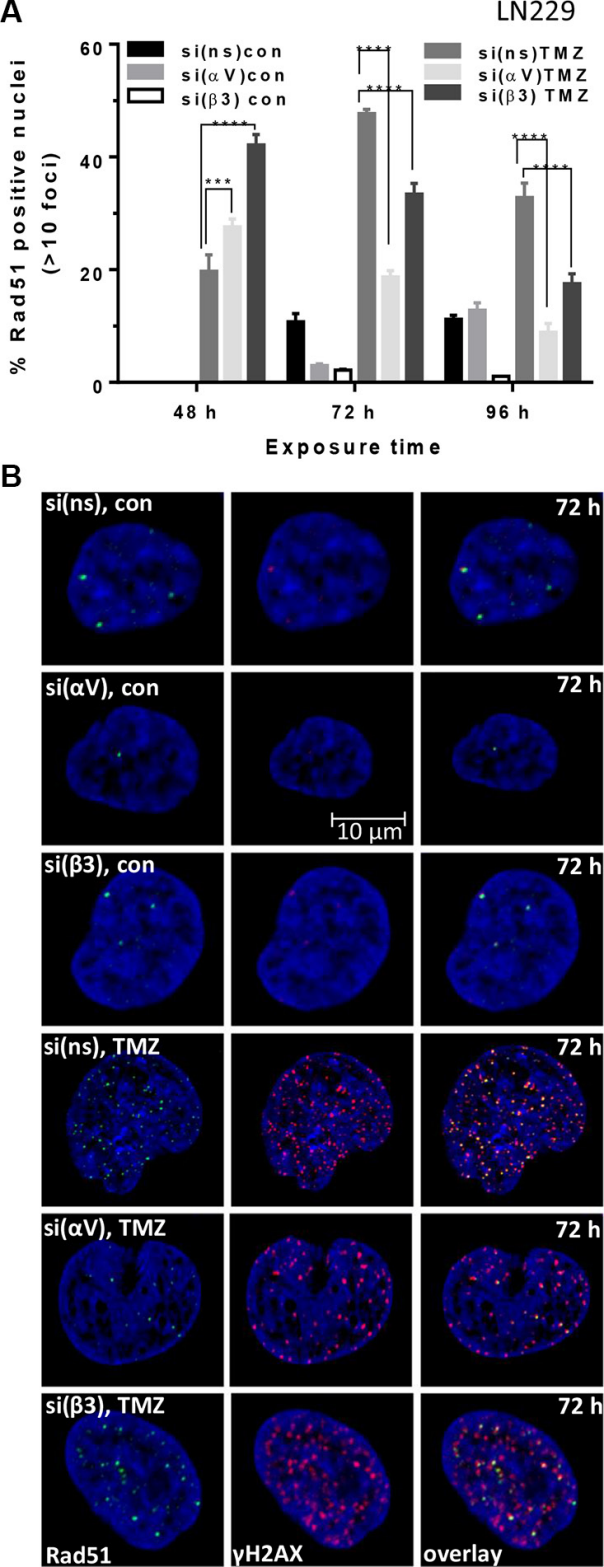
Induction of Rad51 foci upon TMZ/αVβ3 integrin silencing (**A**) LN229 cells were transfected with αV-, β3- or ns-siRNA and exposed to 100 μM TMZ. At indicated time points, the cells were stained for Rad51 and γH2AX. Counterstaining of the nuclei was performed using TO-PRO-3. The data are the mean of two independent experiments in duplicates ± SD. *p ≤ 0.05 significant, **p ≤ 0.01 very significant, ***p ≤ 0.005 highly significant, ****p ≤ 0.001 most significant. n.s., not significant. (**B**) The representative nuclei at 72 h upon TMZ exposure showing co-localization (overlay) of Rad51 foci (green staining) and γH2AX foci (red staining). The nuclei were stained with TO-PRO-3 (blue staining).

Since increased frequency of γH2AX foci pointed to a reduced DSB repair, we examined whether and to what extent the number of Rad51 foci is modulated by silencing of αV or β3 upon TMZ exposure. The data shows that the number of Rad51 foci constantly declined during the exposure time (48–96 h, Figure 5A) whereas the γH2AX foci remained at high level, indicating that HR was compromised. The number of Rad51 foci in LN229 cells after αV or β3 knockdown was dramatically reduced 96 h after TMZ exposure as compared to the number of foci at 48 h. In contrast, the number of Rad51 foci in cells transfected with control-siRNA increased with time and was higher at 72 and 96 h in comparison to 48 h upon exposure to TMZ (see Figure 5A). Whereas all Rad51 foci co-localized with γH2AX foci in control-siRNA transfected cells (indicating ongoing HR) upon integrin αV and β3 knockdown, a lot of γH2AX foci did not co-localize with Rad51, indicating persistent DSBs (Figure 5B). The results were supported by a significant reduction of HR efficiency in LN229 cells transfected with β3-siRNA and treated with TMZ (Figure 4B, for qPCR melting curves, see Supplementary Figure S9).

### Reduction in the number of Rad51 foci is a consequence of αVβ3 integrin silencing

**Figure 6:**
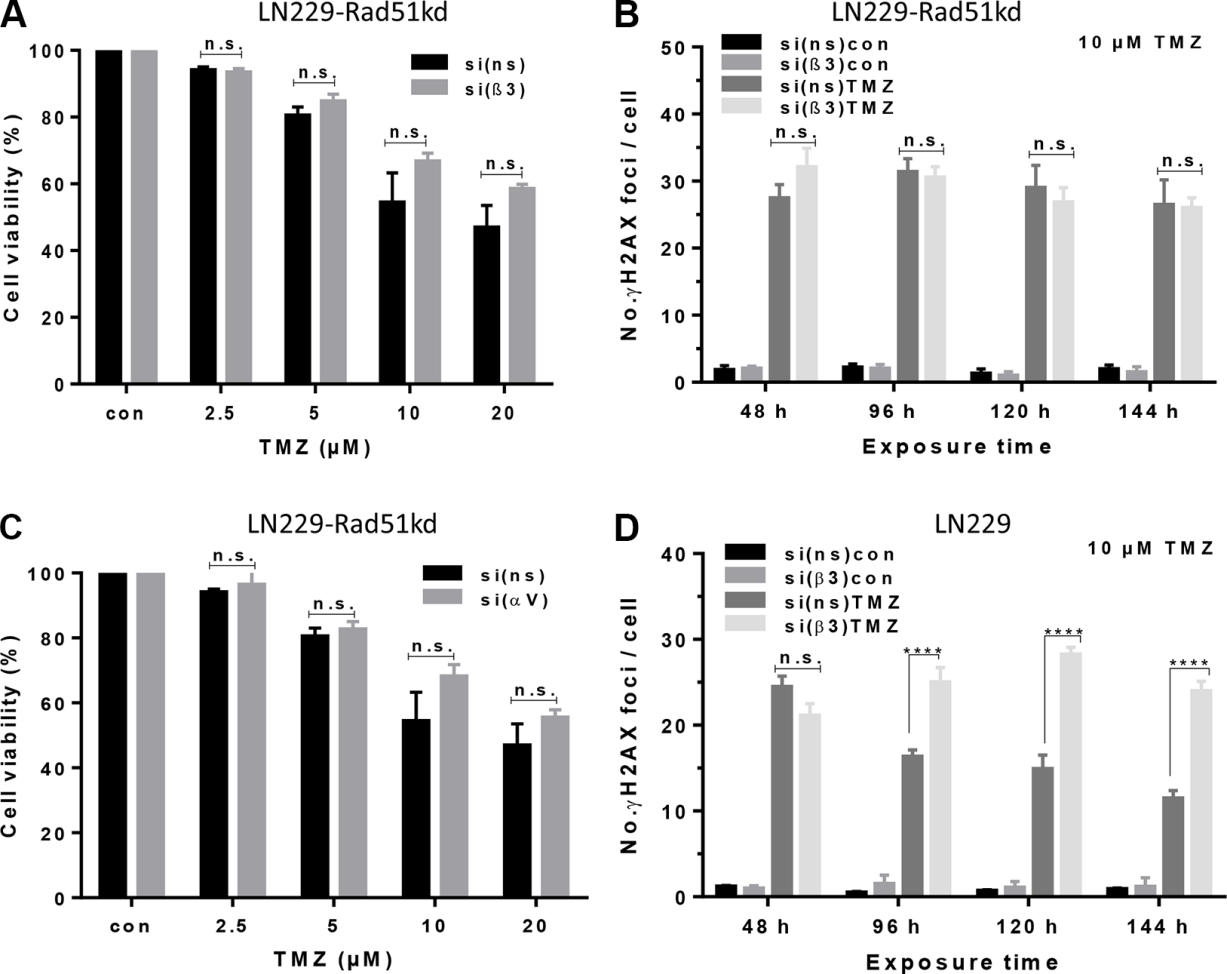
Determination of cell viability and induction of γH2AX foci in Rad51 knockdown cells upon TMZ/αVβ3 integrin silencing (**A**, **B**) LN229-Rad51kd cells were transfected with αV-, β3-, or ns-siRNA and 48 h later exposed to TMZ (2.5–20 μM) for determination of cell viability in the metabolic MTT assay. (**C**) Time-dependent induction of yH2AX in LN229-Rad51kd cells transfected with β3-or ns-siRNA, non-exposed or exposed to 10 μM TMZ. (**D**) Time-dependent induction of γH2AX foci in LN229 cells transfected with β3- or ns-siRNA, non-exposed or exposed to 10 μM TMZ. The data are the mean of two independent experiments in duplicates ± SD. *p ≤ 0.05 significant, **p ≤ 0.01 very significant, ***p ≤ 0.005 highly significant, ****p ≤ 0.001 most significant. n.s., not significant.

In order to examine whether the reduced number of Rad51 foci is a consequence of integrin β3 silencing, we utilized a cell clone with stable Rad51 knockdown (LN229-Rad51kd), which was shown to be hypersensitive to TMZ [23]. In this clone, the integrin subunit β3 or αV was silenced and cells were treated with a low TMZ concentration (2.5–20 μM). As shown in Figure 6A–6B, neither silencing of β3 nor αV had impact on TMZ-induced cytotoxicity in the LN229-Rad51kd clone. There was also no difference in the induction of γH2AX foci in the LN229-Rad51kd clone upon β3 knockdown (Figure 6C). Conversely, the parental LN229 cells transfected with control-siRNA and exposed to 10 μM TMZ were able to significantly repair DSBs, whereas the cells transfected with integrin β3-siRNA were unable to do so, even at this low TMZ concentration (Figure 6D). Collectively, the data indicate that the increased TMZ cytotoxicity upon αVβ3 knockdown is due to a reduced Rad51-dependent repair of DSBs.

### Modulation of integrin signaling kinases and Rad51 expression by β3 silencing and TMZ

**Figure 7:**
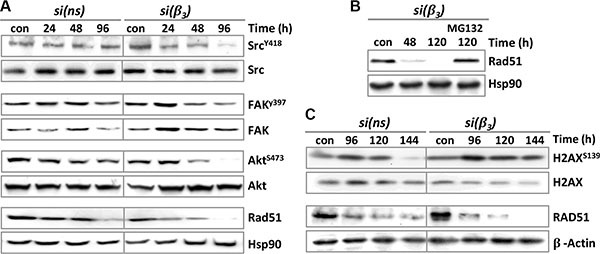
Expression of integrin signaling kinases, DNA repair proteins and apoptotic factors upon TMZ/αVβ3 integrin silencing (**A**, **B**) LN229 cells were transfected with control-siRNA (si(ns)) or β3-siRNA (si(β3)) and 48 h upon transfection exposed to 100 μM TMZ. A. At different time points (24-96 h) upon TMZ exposure, whole-cell extracts were isolated and subjected to western blot analysis of pSrc^Y418^, Src, pFAK^Y397^, FAK, pAkt^S473^, Akt and Rad51. B. The proteasomal inhibitor MG132 (10 μM) was not added or added to the cells 24 h after addition of TMZ, and protein extracts examined for expression of Rad51 (con: TMZ-unexposed cells). Hsp90 was used as loading control. (**C**) LN229 cells were transfected with si(ns) or si(β3) and 48 h later exposed to 100 μM TMZ. The cells were collected at 96-144 h upon TMZ exposure and subjected to western blot analysis of unphosphorylated and phosphorylated H2AX (γH2AX) and Rad51. β-actin was used as loading control.

We also analyzed whether the signaling kinases FAK, Src and ILK are modulated upon β3 knockdown. It is well known that tyrosine 397 of the FAK protein is the main target of autophosphorylation, which leads to the activation of FAK, and renders pFAK (Y397) as high-affinity partner for binding of Src [24]. Similarly, the autophosphorylation at Y418 is required for full catalytic activity of Src [25]. Another catalytic adaptor that directly binds the β1 and β3 integrin cytoplasmic tails is ILK [26]. The data show that pFAK and pSrc expression was reduced 24–96 h upon TMZ treatment (following 48 h β3-siRNA transfection) (Figure 7A), while the expression of ILK was not changed (data not shown), indicating that integrin αVβ3 signals in glioma cells through pFAK and pSrc. In line with this, β3 silencing also resulted in dephosphorylation of the pro-survival kinase Akt (Figure 7A). Most interestingly, TMZ exposure led to a reduction in Rad51 protein expression, but upon β3 silencing the reduction was strongly enhanced and started even earlier i.e. 24 h after TMZ exposure. Reduction of Rad51 was due to proteasomal degradation (Figure 7B) and was most pronounced 120-144 h upon TMZ exposure and paralleled the H2AX phosphorylation (Figure 7C). Since Rad51 degradation was also observed in control-siRNA transfected cells, albeit to a much lower extent, this effect appears to result partially from TMZ itself, but is strongly amplified following β3 silencing (Figure 7A, 7C).

### Modulation of apoptosis pathways by β3 silencing combined with TMZ

**Figure 8:**
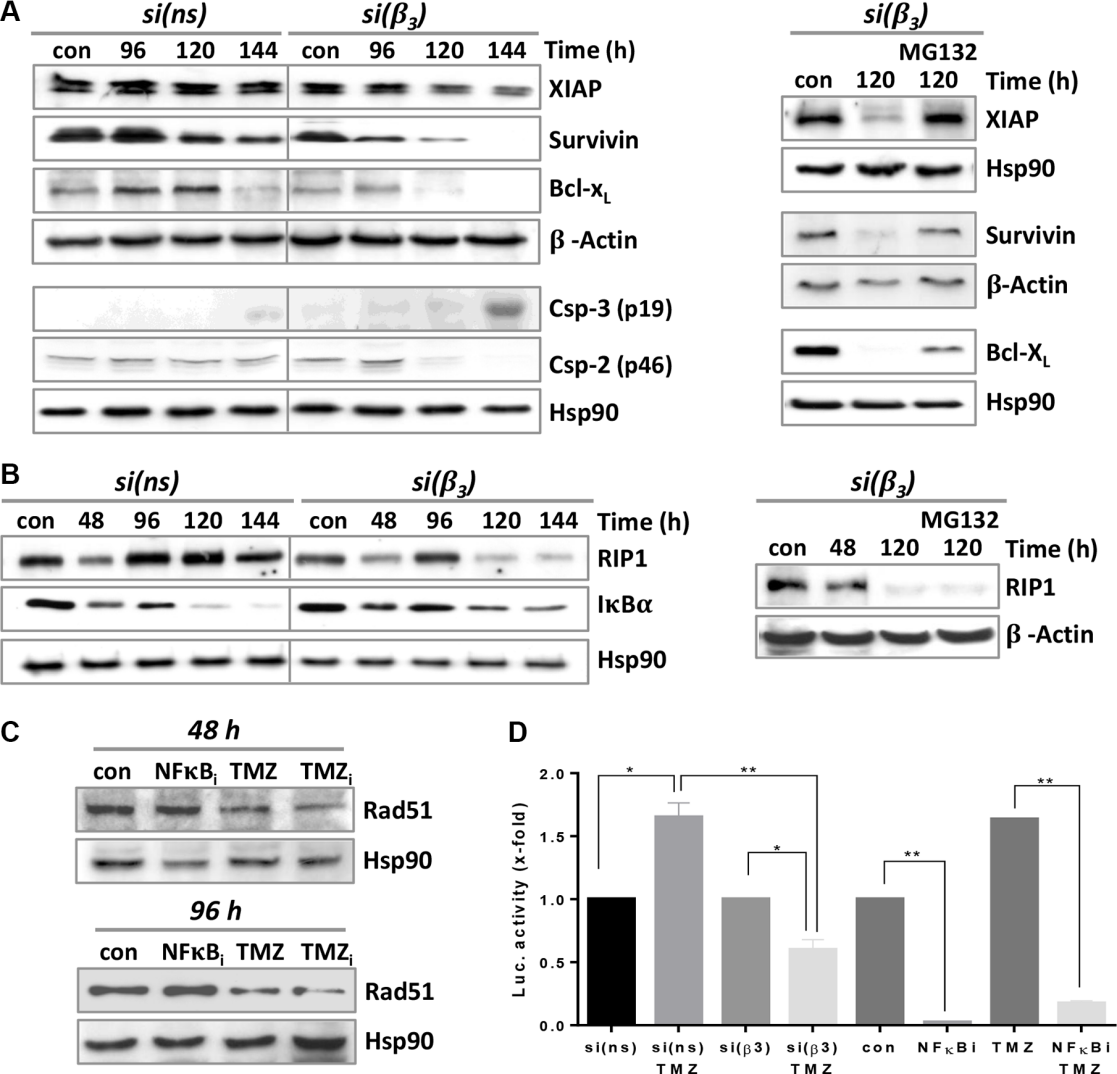
Expression of different pro- and anti-apoptotic factors upon TMZ/αVβ3 integrin silencing (**A**) LN229 cells were transfected with integrin β3- or ns-siRNA and 48 h upon transfection exposed to 100 μM TMZ (left panel) or additionally exposed to MG132 (10 μM) 24 h after begin of TMZ treatment (right panel). The cells were collected 96-144 h after TMZ exposure and subjected to western blot analysis of XIAP, survivin, Bcl-x_L_ and caspase-3/caspase-2 expression. β-actin and Hsp90 were used as loading control. (**B**) LN229 cells were transfected with integrin β3- or ns-siRNA and 48 h later exposed to 100 μM TMZ (left panel) or additionally exposed to MG132 (10 μM) 24 h after begin of TMZ treatment (right panel). The cells were collected at different times upon TMZ exposure and subjected to western blot analysis of RIP1 and IκBα. (**C**) Time-dependent expression of Rad51 after treatment of LN229 cells with NFκB inhibitor (10 μM NFκB_i_), 100 μM TMZ, or a combination of both (TMZ_i_). (**D**) NFκB activity was determined by the dual luciferase reporter assay. Cell lysates were analyzed in triplicates and standardized for transfection using a renilla luciferase construct. The unexposed controls (con, si(ns), si(β3)) were set to 1. NFkB_i_: NFκB inhibitor (10 μM). The data are the mean of two independent experiments in duplicates ± SD. *p ≤ 0.05 significant, **p ≤ 0.01 very significant, ***p ≤ 0.005 highly significant, ****p ≤ 0.001 most significant.

It has been shown that dephosphorylation of Akt might be an important trigger of apoptosis through direct phosphorylation of X-linked inhibitor of apoptosis (XIAP) [27]. Indeed, following Akt dephosphorylation (Figure 7A) XIAP was proteasomally degraded (Figure 8A), which led to a subsequent degradation of survivin and Bcl-x_L_ (Figure 8A). Reduced expression of these anti-apoptotic proteins led to cleavage of caspase-3 and reduction of its downstream target pro-caspase-2 (Figure 8A, left panel). Interestingly, the late activation of caspase-2 (120–144 h) coincided with reduction in RIP1 protein (Figure 8B, left panel), which is one of its putative targets [28]. In line with this, RIP1 kinase, although reduced in its protein level, was not proteasomally degraded (Figure 7B, right panel). RIP1 itself is a crucial factor for activation of NFκB in DNA damage-induced apoptosis [29]. Moreover, IκBα, the inhibitor of NFκB, was found less degraded in β3-siRNA than in control-siRNA transfected cells upon TMZ treatment (Figure 7B), suggesting incomplete activation of NFκB.

### Reduction in NFκB activity by pharmacological inhibition or by integrin silencing reinforces reduction in Rad51 upon TMZ treatment

Recently it was shown that NFκB can directly support HR by binding to the BRCA1-CtiP complex, leading to a more efficient Rad51-dependent repair of DSBs [30]. Therefore, we analyzed whether incomplete activation of NFκB may influence Rad51 expression upon TMZ exposure. To this end, LN229 cells were exposed to TMZ and 24 h later treated with a specific inhibitor of NFκB. As shown in Figure 8C, 96 h upon TMZ exposure Rad51 protein expression was reduced, but the effect was significantly more pronounced following NFκB inhibition. In line with this, the reporter assay showed a strongly reduced NFκB activity upon TMZ/β3 silencing (see Figure 8D). TMZ itself and TMZ/control-siRNA elevated NFκB activity, as reported [31]. Altogether, the data indicates the incomplete activation of NFκB observed upon TMZ/β3 silencing could at late time points additionally decrease Rad51 expression and thus suppress HR activity.

### Verification of *in vitro* data in xenograft model *in vivo*

**Figure 9:**
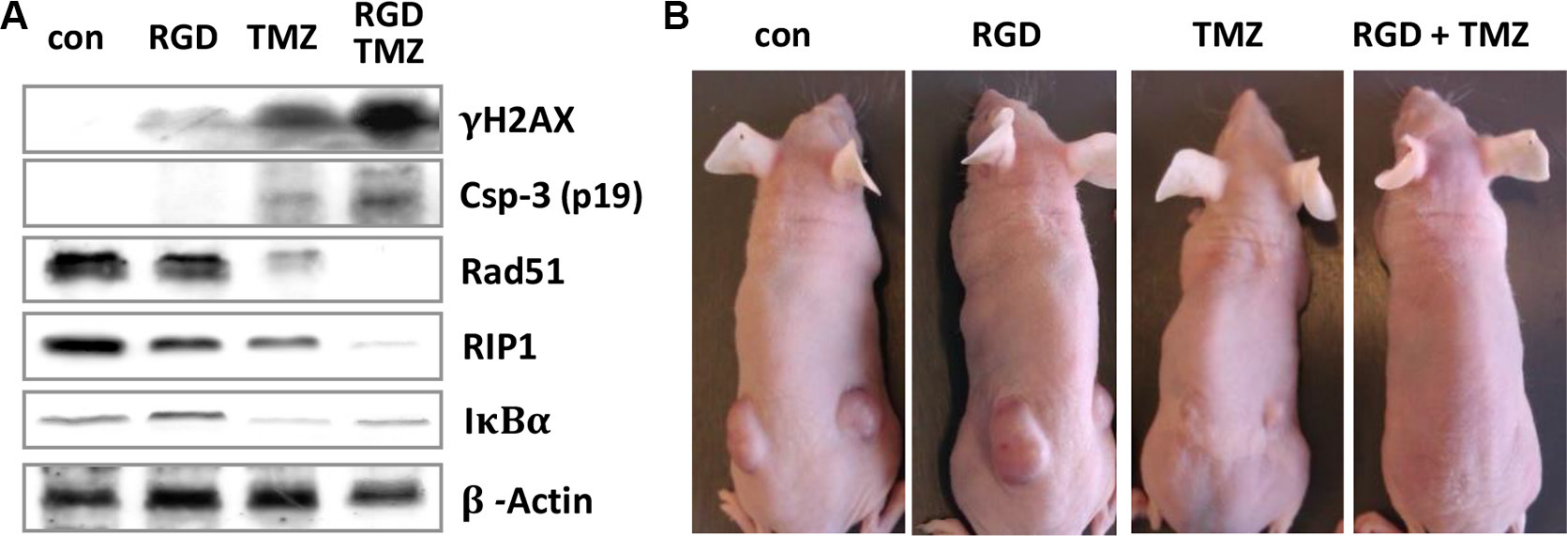
Expression of DNA repair and apoptotic factors in mice xenografts (**A**) Nude mice (Balb/c nu/nu, two animals per each group) with U87MG xenografts were left untreated (con) or intraperitoneally injected with cyclo-RGD integrin antagonist (RGD), TMZ, or a combination of both (RGD+TMZ). After regression time of three weeks, tumors were removed, proteins extracted and subjected to western blot analysis of γH2AX, Rad51, RIP1, IκBα and cleaved caspase-3. β-actin was used as loading control. (**B**) Representative images of the nude mice, showing regression of tumors in TMZ- and RGD/TMZ-treated animals.

To verify the molecular mechanism *in vivo* and to demonstrate that inhibition of integrin signaling has impact on DNA repair and the cytotoxic response of glioma cells to TMZ, we treated nude mice bearing U87MG tumor xenografts with the cyclo-RGD inhibitor and challenged them with TMZ. As shown in Figure 9A, expression of γH2AX and cleaved caspase-3, as well as reduction in Rad51 and RIP1 expression and sustained level of IκBα expression were significantly more enhanced in tumor extracts from animals treated with the inhibitor and TMZ, in comparison to non-treated mice and mice treated with the RGD inhibitor or TMZ only, revealing the same molecular mechanism to occur also *in vivo*. Similar to *in vitro* results, inhibition of αVβ3 by the RGD inhibitor enhanced the TMZ response and furthermore triggered tumor regression (examples are shown in Figure 9B).

### Effect of integrin αV silencing on migration of glioma cells

**Figure 10:**
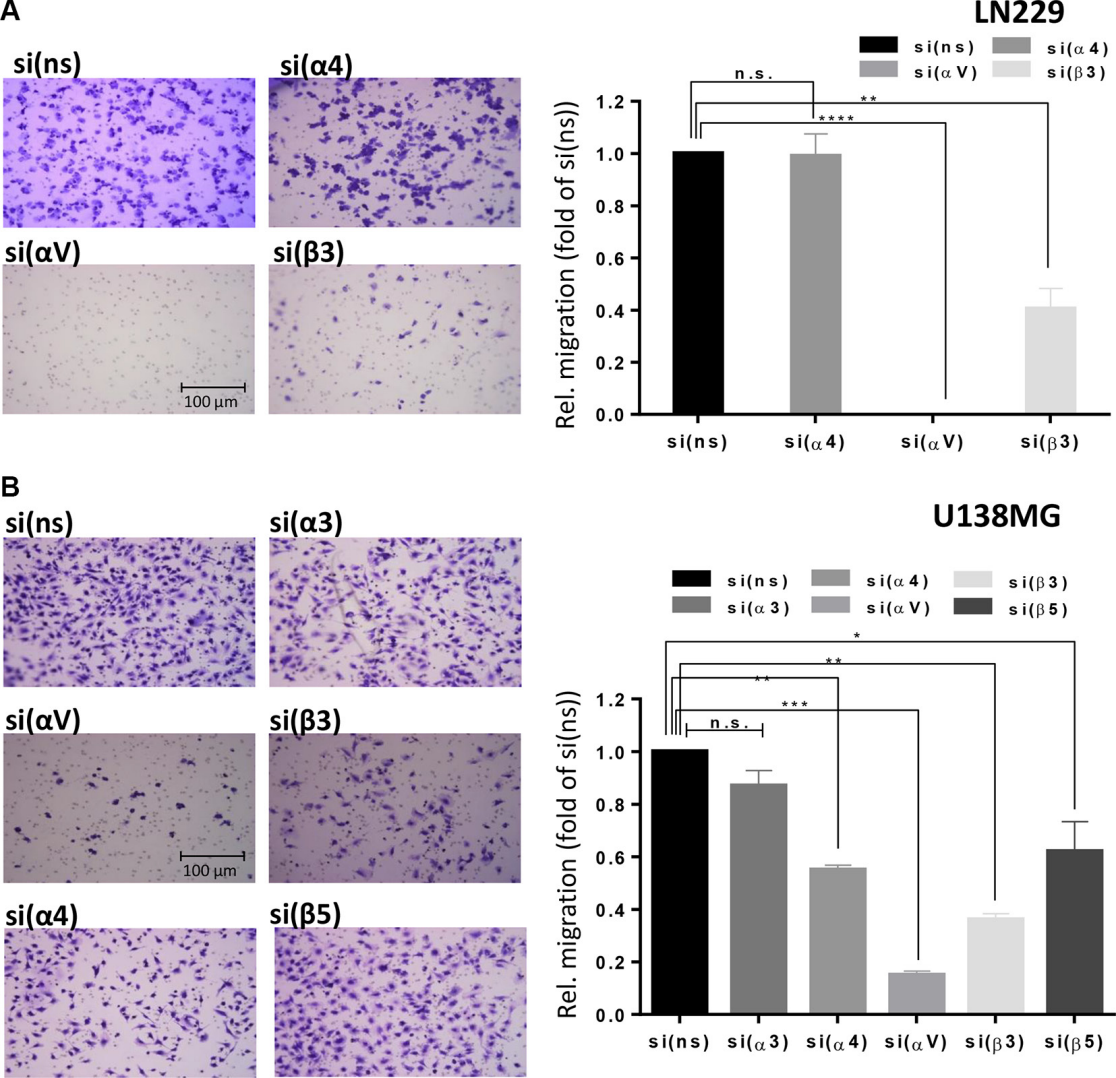
Integrin subunit knockdown decreases migration Cell migration was determined in (**A**) LN229 cells upon transfection with control-, α4-, αv- or β3-siRNA and (B) U138MG cells upon transfection with control-, α3-, α4-, αv-, β5- or β3-siRNA. 48 h after transfection, the cells were starved for 24 h and cell migration was measured in Boyden chambers. After 22 h, cells on the underside of the membrane were stained with crystal violet, photographed and counted. Averages of five microscope fields ± SD are shown. *p ≤ 0.05 significant, **p ≤ 0.01 very significant, ***p ≤ 0.005 highly significant, ****p ≤ 0.001 most significant.

In order to substantiate the decisive role of integrins αV as feasible targets not only for sensitization to alkylation-based glioma therapy in terms of induction of apoptosis but also to combat metastasis, we conducted migration assays. We observed that upon knockdown of different integrins in LN229 (Figure 10A) and U138MG (Figure 10B) cells migration was significantly decreased, but the most dramatic effect was observed in LN229 cells upon αV knockdown. A similar highly potent effect of integrin αV knockdown was observed in U138MG cells, reflecting the involvement of both αVβ3 and αVβ5 in migration. In U138MG cells the β3 knockdown inhibited migration significantly more efficient than knockdown of β5, suggesting again that integrin αVβ3 is a potential therapeutic target for inhibition of metastasis. The knockdown of α4 in U138MG cells significantly inhibited migration but in LN229 cells did not affect migration at all.

## DISCUSSION

Some integrin-targeting strategies like monoclonal antibodies and small molecules as CGT, which was shown to inhibit the activation of integrins αVβ3 and αVβ5 in the orthotopic brain model thereby inhibiting angiogenesis [11] have been tested in clinical trials [6]. It has been shown that αV antagonists inhibit cancer growth independent of the anti-angiogenic effect [32]. Co-delivery of siRNAs with anticancer agents provides promising option for an improved chemotherapeutic effect. Various nanocarriers, which are not devoid of limitations, have been developed to deliver siRNA and drugs. The major challenge for siRNA-based therapeutics includes minimizing the potential off-target effects and controlling the specificity of the siRNA. However, it is anticipated that the research on combination delivery of siRNA and chemotherapeutics will progress with increase in the knowledge and innovative delivery strategies [33].

Although in a phase I/II study CGT had a beneficial effect for patients having tumors with MGMT promoter methylation [13], this positive curative effect was not confirmed in the phase III (CENTRIC) study [14]. In our opinion, the failure of the study could be explained by the CGT pharmacokinetics. In the CENTRIC study, CGT was applied as anti-angiogenic drug and probably poorly reached the tumor. In this context, it is important to note that the effectiveness of anti-integrin drugs was critically assessed before [34]. Thus, it was shown that a too low antagonist concentration instead of inhibiting, promotes angiogenesis [35]. Another reason for the failure might be the opposite action of radiotherapy and CGT. It was shown that IR induces expression of αVβ3 in human endothelial [32] and glioma cells [36]. If this is the case, concurrent integrin antagonists may be beneficial only in a TMZ mono-therapy setting. In spite of the negative outcome of the CENTRIC and CORE study, the data suggests that integrins remain an attractive target for cancer therapy.

Based on the data obtained in a panel of ten glioblastoma cell lines and the fact that αVβ3 represents the most frequently expressed integrin heterodimer on malignant gliomas [37], we focused on its impact on TMZ-induced cytotoxicity and repair *in vitro* and *in vivo*. Moreover, the expression of αVβ3 in tumor cells and vessels, but not of other αV integrins, was shown to be dependent on tumor grade, and αVβ3 expression in tumor cells may have a prognostic impact [38]. We could show that silencing of all of the respective integrin subunits (αV, α3, α4, β3 and β5) sensitized glioblastoma cell lines to TMZ but the silencing of αVβ3 was the most powerful. Moreover, cytotoxicity of TMZ was also enhanced in glioblastoma cell lines using a cyclo-RGD antagonist, which inhibits integrin αVβ3 comparably to CGT but needs much higher concentrations to antagonize αVβ5. Since the applied concentrations were rather low, we suppose that the sensitizing effect of the inhibitor is primarily attributed to silencing of αVβ3. The decisive role of integrins αV for glioma therapy response was corroborated by decreased migration after their knockdown in LN229 and U138MG cells, thus indicating decreased metastatic potential. In LN229 cells the effect of integrin αV knockdown was the most dominant, indicating that not only integrin αVβ3, but very likely also integrin αVβ1 is involved in cell migration. A similar highly potent effect of integrin αV knockdown was observed in U138MG cells, reflecting the involvement of several αV integrin heterodimers in migration, i.e. at least αVβ3 and αVβ5. In U138MG cells the β3 knockdown inhibited migration significantly more efficient than knockdown of β5, suggesting again that integrin αVβ3 is not only a potential therapeutic target for sensitization to TMZ but also for inhibition of metastasis.

Our results show that silencing of the integrins α3β1, α4β1, αVβ3 and αVβ5 strongly enhanced the TMZ-induced formation of DSBs, as determined by γH2AX foci induction. This was accompanied by a significantly reduced expression of the Rad51 protein due to proteasomal degradation, leading to a decline in the Rad51 foci level. Rad51 represents a rate-limiting factor for repair of TMZ-induced DSBs by HR [23]. Indeed, LN229 cells showed a decreased HR efficiency, indicating that decreased expression of Rad51 abrogates the repair of DSBs. This conclusion is further supported by the finding that β3 silencing was neither able to further sensitize Rad51 knockdown cells to low-dose TMZ nor to enhance the number of TMZ-induced γH2AX foci. Thus suppression of DSB repair upon β3 silencing equals the effect of a direct Rad51 inhibition which is in line with literature data [39]. As expected, inhibition of αVβ3 by the cyclo-RGD antagonist in combination with TMZ in the glioma xenograft model, led to pronounced tumor regression and showed consistently reinforced γH2AX phosphorylation and caspase-3 cleavage, strongly reduced Rad51 and RIP1 expression, and revealed a persistent IκBα expression in tumors *in situ* compared to mice treated with TMZ alone. Thus, the data obtained in cell culture and *in vivo* reveals a very similar molecular mechanism, showing that inhibition of αVβ3 affects the DNA repair capacity and ameliorates the cytotoxic response of glioma cells to TMZ.

The proteasomal degradation of Rad51 seems to be an important mechanism in sensitizing cancer cells by suppressing HR [40, 41]. In our hands, the efficiency of non-homologous end-joining upon TMZ/β3 silencing was not affected, as shown by the radioactive DNA-PKcs activity assay (Supplementary Figure S10), contrary to the published data for IR/β1 silencing [42]. Upon TMZ/β3 silencing we observed a modest slow-down in progression of cells in all phases of the cell cycle, as already mentioned. Nevertheless, we cannot exclude that this moderate arrest in S- and G2-phase contributes to the decreased Rad51 levels and impairment of HR.

Integrin-mediated adhesion modulates the expression of proteins like PI3K/Akt, Bad, Bcl-2, Bcl- x_L_, survivin, XIAP, p27^Kip^, and activation of MAP kinase pathways, activation of PARP and NFκB [43]. Thus, we further analyzed which signaling pathways and apoptotic factors are altered upon integrin silencing. Our data revealed that integrin αVβ3 transmits signals through pFAK, pSrc and pAkt, which is in line with results showing that Src silencing enhanced the cytotoxic effect of TMZ in LN229 cells [44]. The same inhibition of FAK/Src/Akt in glioma cells was also shown upon CGT [45]. In our experiments β3 silencing led to reduction in phosphorylation and expression of XIAP, which is presumably associated with the observed inactivation (dephosphorylation) of Akt [27]. The reduced expression of XIAP was also a result of proteasomal degradation and was associated with reduced expression of survivin, an essential mitotic and anti-apoptotic protein, which can be marked for degradation by the E3 ubiqutin ligase activity of XIAP [46]. Furthermore, we observed a reduced expression of the anti-apoptotic Bcl-x_L_ protein, which might be explained through abrogation of Bad phosphorylation by Akt. In this case hypophosphorylated Bad could deliberate Bcl-x_L_ from the complex allowing Bcl-x_L_ degradation [27]. TMZ/β3 silencing finally led to activation of caspase-3 and a late decline in the level of pro-caspase-2, a known downstream target of caspase-3 [47]. The activation of caspase-2 coincided with reduction in RIP1, which is a crucial factor for activation of NFκB in DNA damage-induced apoptosis [29, 48]. Since reduction in RIP1 expression could not be inhibited by the MG132 proteasomal inhibitor, it might be a result of a direct cleavage by caspase-2, for which RIP1 was postulated to be a putative target [28]. Involvement of caspase-8 in RIP1 cleavage could be excluded since caspase-8 was not activated in our model (data not shown). In addition to reduced RIP1 expression at late time points, IκBα was stabilized upon TMZ/β3 silencing. Both, down-regulation of RIP1 and enhanced stability of IκBα may lead to incomplete activation of NFκB at late time points, as supported by reduced NFκB activity in the reporter assay. NFκB can act as a pro-survival transcription factor [49–51]. Upon TMZ/β3 silencing, however, NFκB is unlikely to be involved in the transcriptional regulation of *Bcl-x*_L_*, survivin and XIAP*, since these factors were degraded by the proteasome. Besides being a transcription factor, NFκB can play a direct role in HR repair, stimulating Rad51 activity. In this case, NFκB regulates repair of DSBs in conjunction with BRCA1-CtiP complex [30] which might also be the case upon TMZ/β3 silencing. This assumption was further supported by the observation that pharmacological NFκB inhibition combined with TMZ accelerated the Rad51 degradation, in comparison to TMZ alone (see Figure 7C). Thereby NFκB inhibition recapitulates the effect of β3 silencing on the Rad51 expression. However since Rad51 degradation was observed prior to the impairment of the NFκB function following β3 silencing, reduced NFκB activity cannot be the initial trigger. It is conceivable, however, that it suppresses Rad51 expression and HR activity at late time points, thereby amplifying the TMZ cytotoxicity.

Overall the present study shows that inhibition of the integrin αVβ3 sensitizes glioma cells to TMZ, altering multiple molecular pathways like DNA repair, signaling and apoptosis. Thus αVβ3 targeting combined with TMZ is a valuable approach to improve TMZ therapy of malignant gliomas.

## MATERIALS AND METHODS

### Ethics statement

Investigation has been conducted in accordance with the ethical standards and according to the Declaration of Helsinki and according to national and international guidelines and has been approved by the authors’ institutional review board.

### Cell culture

The human glioblastoma cell line U87MG was purchased from Cell Line Service (Eppelheim, Germany), and the glioblastoma cell line LN229 was obtained from LGC Standards (Wesel am Rhein, Germany). The glioblastoma cell lines LN308, LN319, LN428, LN18, D247MG and U138MG were kindly provided by Prof. Weller (Laboratory of Molecular Neuro-Oncology, University Hospital and University of Zurich, Switzerland) and were characterized [20]. pSUPER-Rad51sh transfected LN229 cells (LN229-Rad51kd) were previously described [23]. The cell lines were grown in Dulbecco's modified Eagle's medium with 10% fetal bovine serum (Invitrogen) at 37°C, 7% CO_2_.

### Subcutaneous xenografts in nude mice

To induce subcutaneous xenografts, U87MG cells (2.5 × 10^6^) were injected into both flanks of 8-week old immunodeficient mice (Balb/c nu/nu). When tumors reached a suitable size (22 mm^3^), animals were separated into four gender-mixed groups (2 mice per group): 1) Control group (1/4 DMSO to 3/4 PBS i.p.), 2) TMZ group (200 mg/kg body weight in DMSO/PBS i.p.), 3) RGD group (cyclo-RGD peptide, ∼500 μg i.p., administered only once), 4) RGD + TMZ group (∼500 μg RGD i.p. two hours prior to TMZ injection, 200 mg/kg body weight i.p.). Three weeks later the mice were sacrificed and tumor tissue disintegrated, as described [52]. We deliberately kept the number of animals at minimum since our goal was to verify the molecular mechanism, i.e. to prove expression of key repair and apoptotic players in regressed tumors and not to show regression on its own; the fact that under those circumstances (TMZ alone or TMZ+RGD) tumors regress has been repeatedly shown.

### Drugs and chemicals

TMZ was dissolved in DMSO (35 mM stock) and stored in aliquots at −80°C. The cyclo-RGD peptide (BML- AM100-0001, Enzo Life Sciences) was diluted in PBS. The proteasomal inhibitor MG132 and the NFκB inhibitor (Merck Millipore) were dissolved in DMSO (10 mM and 30.4 mM stock, respectively), stored in aliquots at −20°C and applied at 10 μM.

### Determination of integrin expression by flow cytometry

Membrane fluorescence staining for αVβ3, αVβ5, α3β1 and α4β1 was performed using primary monoclonal antibodies against αVβ3, αVβ5, α3β1 and α4 (see Supplementary Table S1). The binding of the unlabeled antibodies was visualized by the secondary goat Alexa Fluor^®^ 488-coupled anti-mouse antibody (see Supplementary Table S1). Acquisition was conducted using FACS*Calibur* (BD Biosciences).

### Transfection of small interfering RNA (siRNA)

For silencing of integrin subunits β3, β5, αV, α3 and α4, the predesigned integrin-specific siRNA sequences (Silencer Select Predesigned siRNA; Ambion; siRNA ID# s7581 (si(β3)), s7591 (si(β5)), s7568 (si(αV)), s7543 (si(α3)), s7544 (si(α4)) and control human non-silencing siRNA (si(ns)) (Silencer Select Predesigned siRNA Negative Control #1 siRNA; Ambion) were used. The transfections of siRNAs were performed using Lipofectamine RNAiMAX Reagent (Invitrogen).

### Determination of cell death

MTT and colony formation assay were conducted as described [52]. For drug-induced apoptosis and cell cycle distribution cells were fixed in 70% ethanol and treated with DNase-free RNase and stained with propidium iodide, as described [53]. Alternatively, to distinguish apoptosis from necrosis, annexin V-FITC/propidium iodide assay was conducted [53]. Frequency of apoptosis (subG1 and annexin V fractions) was determined by flow cytometry (FACSCanto II, BD Biosciences) using the Cell Quest Pro software. The caspase-3/7 activity was performed according to the manufacturer's protocol (Caspase-Glo 3/7 Assay, Promega). In brief, integrin-specific siRNA-transfected cells were re-seeded into half white-bottom MTP (100 μL maximum volume), or alternatively directly seeded into MTP (500-1000 cells per well), and exposed to anticancer agents. After appropriate time for induction of apoptosis (120 h), the substrate was added directly to the medium with cells. One hour later, induction signal was determined fluorometrically. Acquired values were quantified as relative activity (x-fold induction) in TMZ-exposed compared to non-exposed cells that were set to 1.

### Determination of cell migration

Cell migration was evaluated using Transwell inserts (Corning). Serum starved (24 h) cells were placed in the upper chamber (8 × 10^4^ cells in 0.5 mL DMEM containing 0.1% BSA) and 1 mL of DMEM containing 10% FBS was placed in the lower chamber of 24-well plate. After 22 h in culture, cells on the upper side of the filters were removed with cotton-tipped swabs, the filters were fixed in PFA for 15 min and stained with 1% crystal violet in PBS for 90 min. Cells on the underside of the filters were photographed under light microscope and counted.

### HR activity assay

Efficiency of HR was determined by a qPCR-based HR Assay kit (Norgen Biotek Corporation, ON, Canada), as described [54]. LN229 cells were transfected with siRNA and 18 h later exposed to 100 μM TMZ. 72 h later the cells were transfected with the HR plasmids and 24 h thereafter subjected to isolation of total cellular DNA. Samples were standardized with universal primers, detecting the plasmid backbone for control of transfection efficiency.

### NFκB activity assay

Cells were transfected with the firefly luciferase NFκB-specific reporter gene construct (3 × NFκB-luciferase) [55] and co-transfected with a renilla luciferase reporter as an internal normalization control using Hyperfect Transfection Reagent (Qiagen).

### Preparation of cell extracts and western blot analysis

Whole-cell extracts were prepared by direct lysis with to 95°C heated 1x loading buffer (Roti-Load^®^, Roth), as described [53]. Proteins were separated by SDS-PAGE and transferred onto a nitrocellulose membrane (Amersham) by wet blotting, blocked in 5% non-fat dry milk in TBS-Tween and incubated with specific antibodies. Protein signals were detected using ECL reagent (Pierce). Antibodies used for western blot analysis are specified in the Supplementary Table S1.

### Immunofluorescence

Cells were seeded onto pre-cleaned cover slips in duplicates, transfected with siRNA and/or exposed to TMZ. Cells were washed in PBS and fixed with 4% paraformaldehyde for 15 min at RT. After washing with PBS, cells were incubated with ice-cold methanol for 10 min at −20°C. After three washing steps with PBS, to avoid unspecific binding, the blocking reagent (PBS + 0.25% Triton 100-X + 10% normal goat serum) was added for 1 h. The Rad51/yH2AX staining and evaluation was conducted as described [56]. After washing, nuclei were either stained with TO-PRO-3 (1:1000, 15 min; only for acquisition on LSM), or treated with 10 μL anti-fade medium (Vectashield) with DAPI (only for acquisition with Metafer system). For each treatment sample, 50 cells were analyzed. Microscopy images were screened and captured using Zeiss Axio Imager M1 (Carl Zeiss) supplied with the Metafer4 Software (MetaSystems, Altlussheim, Germany).

### Statistics

The data were evaluated using two way analysis of variance (two-way ANOVA) followed by Bonferroni-correction and were expressed as a mean ± SD. *p ≤ 0.05 was considered statistically significant, **p ≤ 0.01 very significant, ***p ≤ 0.005 highly significant and ****p ≤ 0.001 most significant. Statistical analyses were performed using GraphPad Prism version 6.01 for Windows, GraphPad Software, La Jolla California USA (www.graphpad.com).

## SUPPLEMENTARY MATERIALS FIGURES AND TABLE


