# Supplementary material for: Integrin αVβ3 silencing sensitizes malignant glioma cells to temozolomide by suppression of homologous recombination repair
Source: Oncotarget. 2016 Jul 28;8(17):27754–71. doi: 10.18632/oncotarget.10897 (PMC5438606; doi:10.18632/oncotarget.10897)
Supplement: Supplementary file 1 [file oncotarget-08-27754-s001.pdf]

Integrin αVβ3 silencing sensitizes malignant glioma cells to temozolomide by suppression of homologous recombination repair

Supplementary Materials

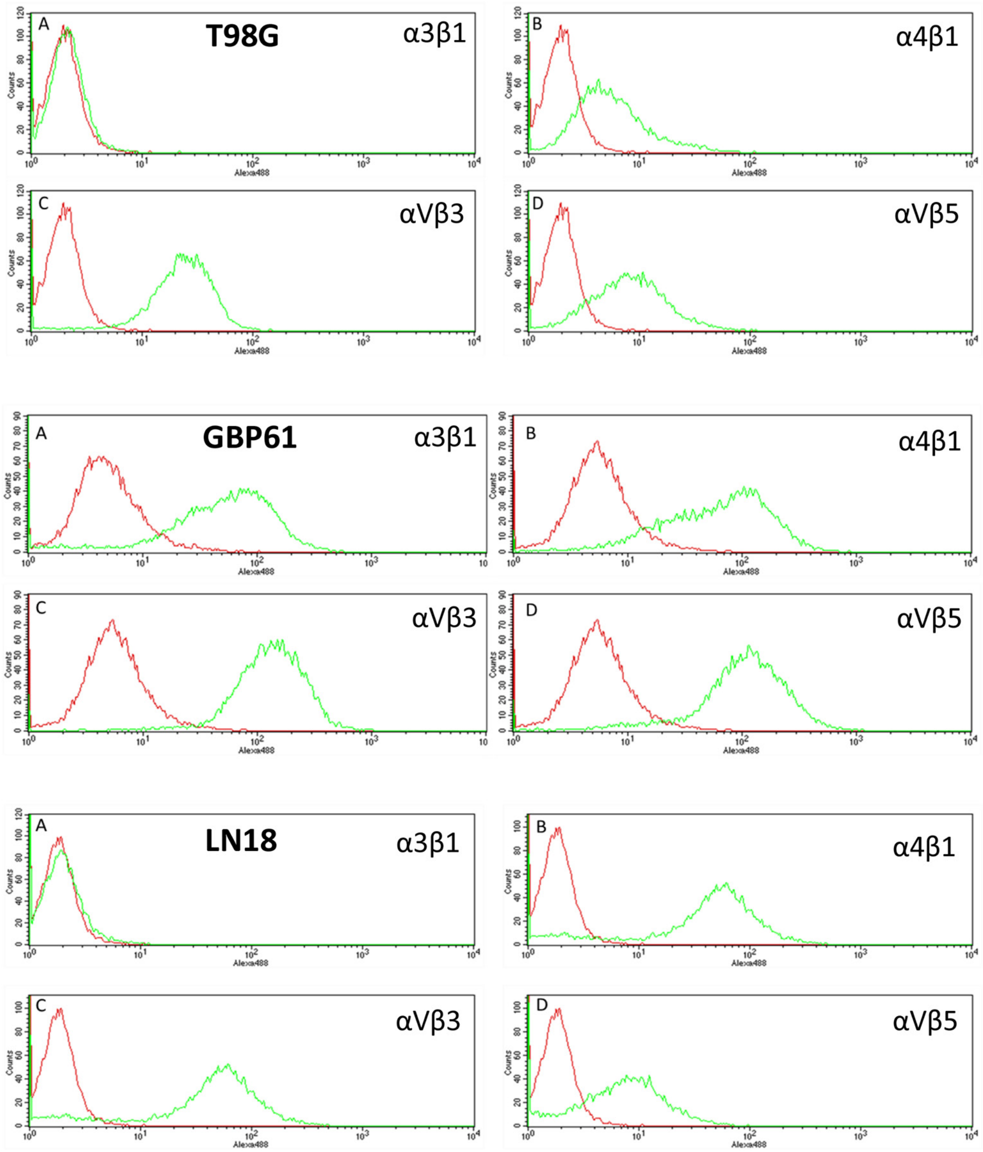

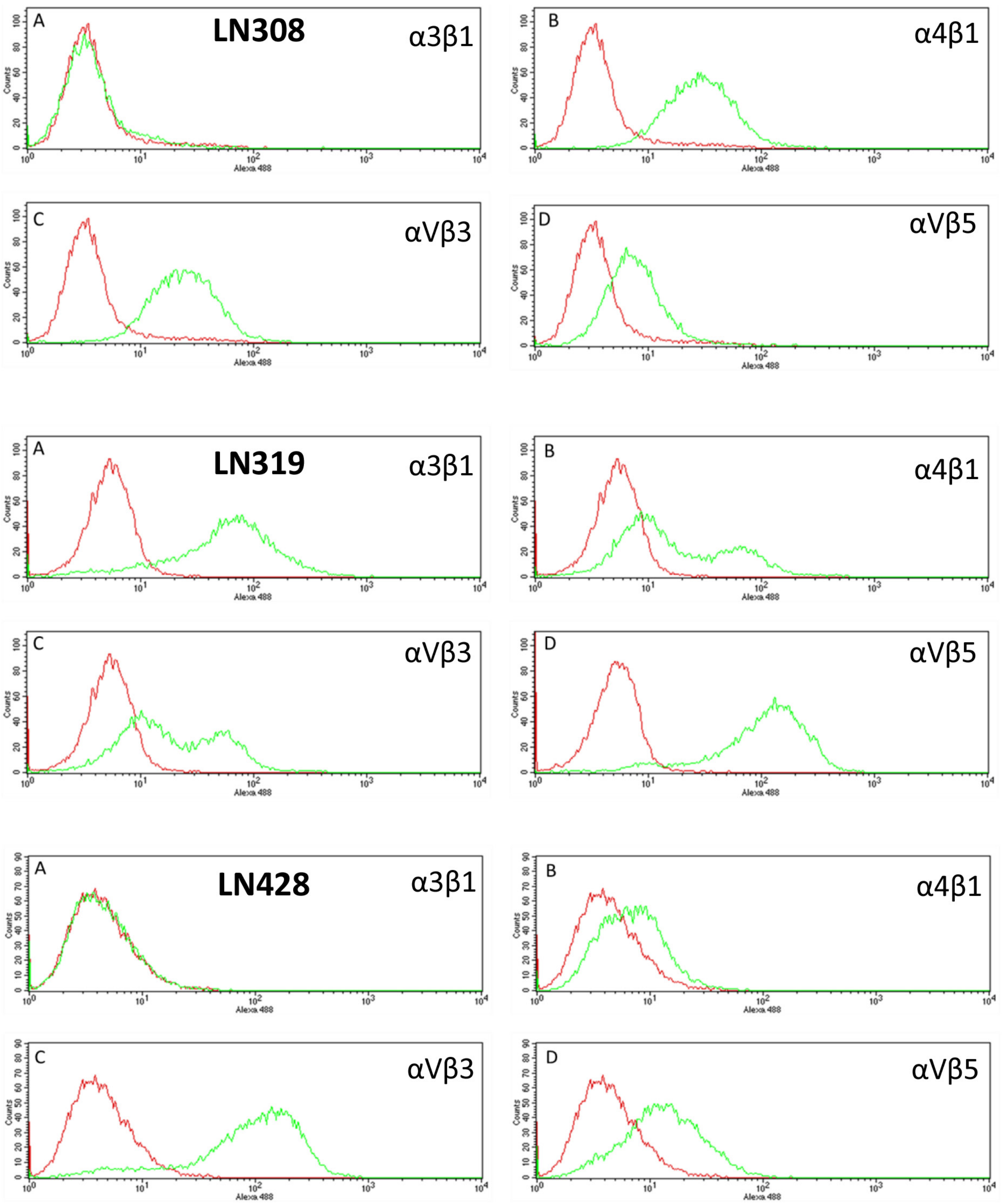
LN308
A
α3β1
B
α4β1
C
αVβ3
D
αVβ5
LN319
A
α3β1
B
α4β1
C
αVβ3
D
αVβ5
LN428
A
α3β1
B
α4β1
C
αVβ3
D
αVβ5

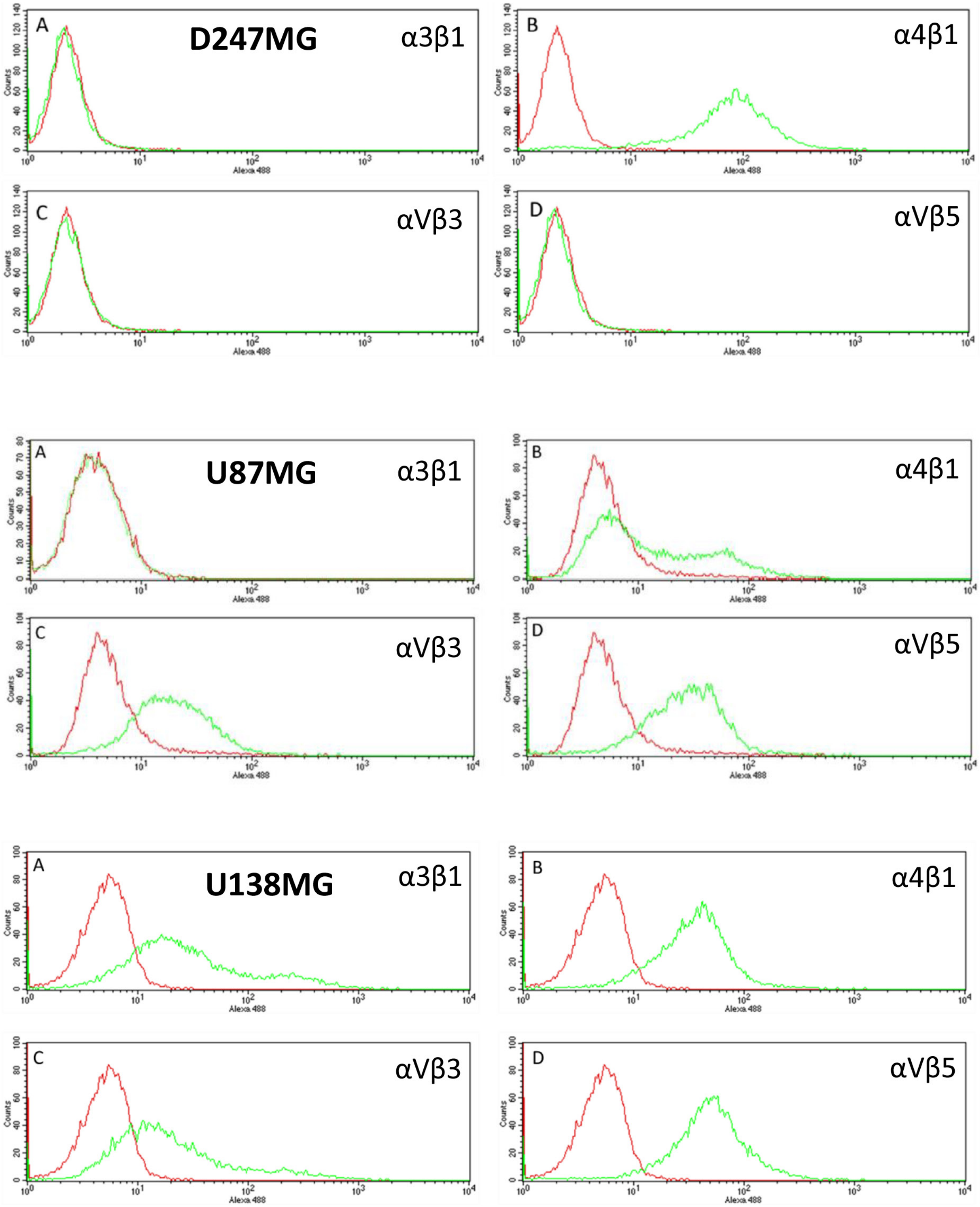

Supplementary Figure S1: Integrin expression in malignant glioma cell lines. Histograms of expression of integrin heterodimers α3β1, α4β1, αVβ3 and αVβ5 in a panel of nine glioma cell lines, as determined by flow cytometry. Red line, no antibodies; green line, specific primary mouse monoclonal antibody plus secondary Alexa Fluor 488-coupled anti-mouse antibody.

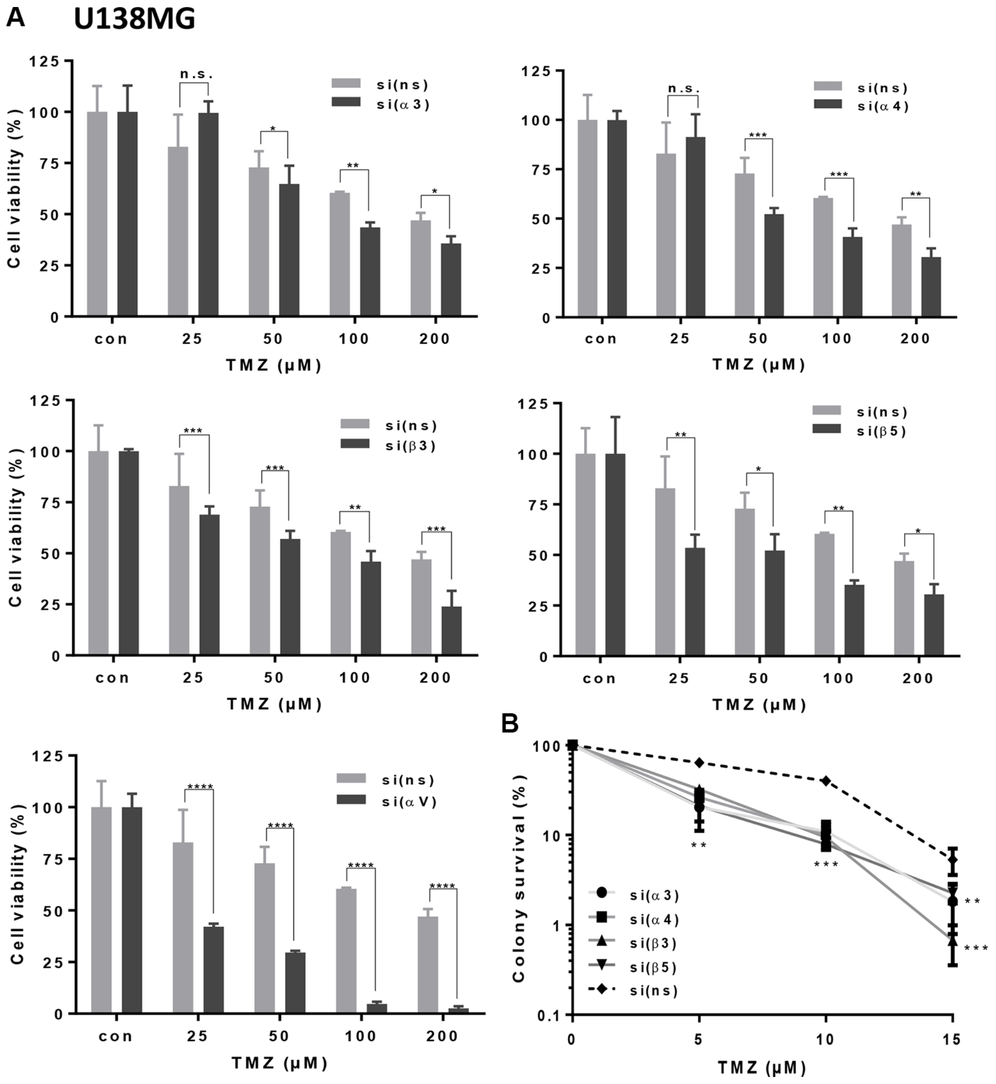

Supplementary Figure S2: Sensitization to TMZ after specific integrin subunit silencing in U138MG cells. (**A**) U138MG cells were transfected with integrin αV-, α3- α4-, β3-, β5- or ns-siRNA (50 nM), 24 h later collected by trypsinization and seeded (1×10^4) for MTT. After another 24 h the cells were treated with increasing concentrations of TMZ, and after 6 days subjected to MTT staining The data are the mean of three independent experiments in quadruplicates ± SD. (**B**) U138MG cells were seeded for colony formation assay (1×10^3 cells per 6-cm plate), 24 h later transfected with integrin α3-, α4-, β3-, β5- or ns-siRNA (50 nM) and exposed to TMZ (5–15 µM). 14 days later the visible colonies were fixed, stained and counted. The data are the mean of three independent experiments ± SD

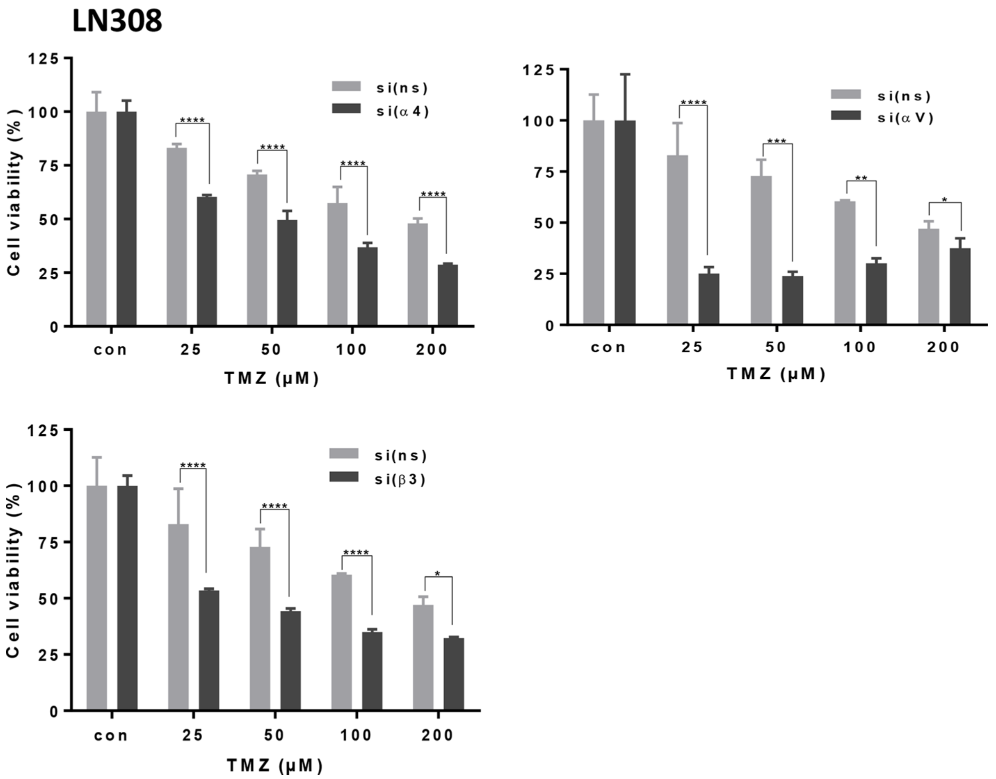

Supplementary Figure S3: Sensitization to TMZ after specific integrin silencing in LN308 cells. LN308 cells were transfected with integrin α4-, αV-, β3- or ns-siRNA (50 nM), 24 h later collected by trypsinization and seeded (1×10^4) for cell viability assay (MTT). After another 24 h the cells were treated with increasing concentrations of TMZ, and 6 days later the MTT staining was conducted according to the protocol. The data are the mean of three independent experiments in quadruplicates ± SD.

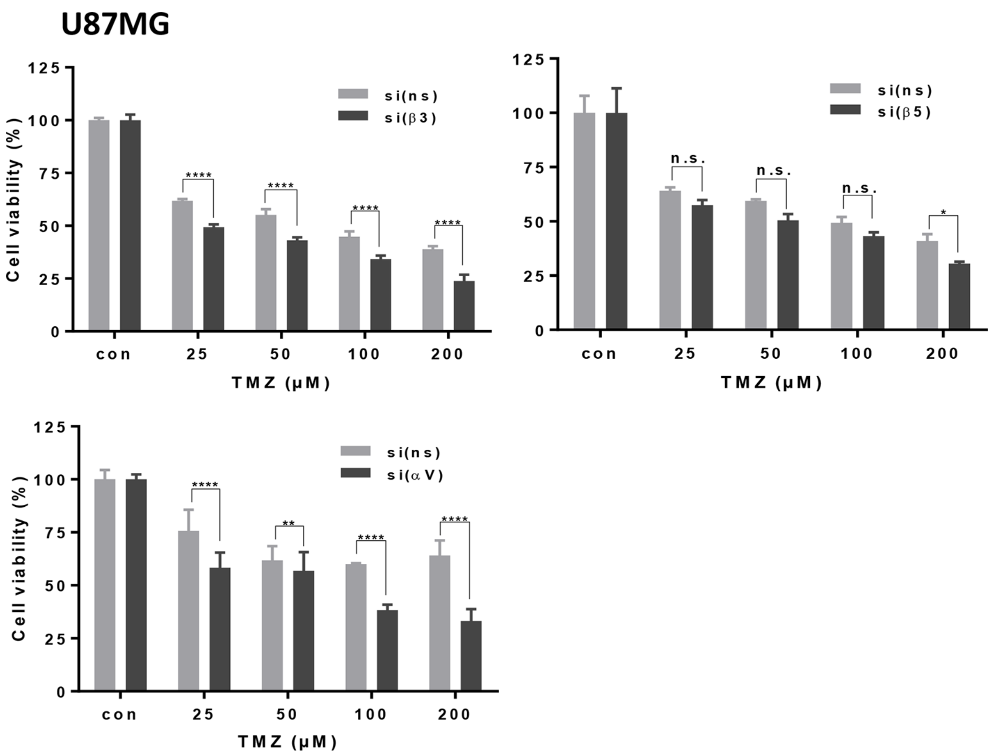

Supplementary Figure S4: Sensitization to TMZ after specific integrin silencing in U87MG cells. U87MG cells were transfected with integrin β3-, β5-, αV- or ns-siRNA (50 nM), 24 h later collected by trypsinization and seeded (1×10^4) for cell viability assay (MTT). After another 24 h the cells were treated with increasing concentrations of TMZ, and 6 days later the MTT staining was conducted according to the protocol. The data are the mean of three independent experiments in quadruplicates ± SD.

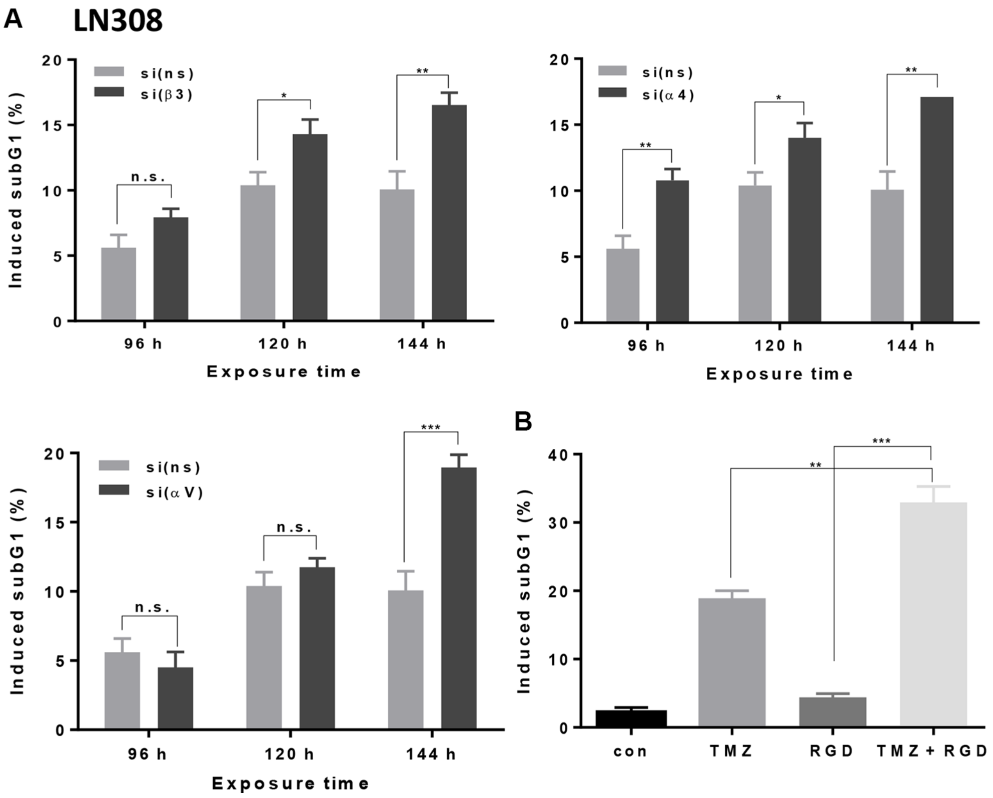

Supplementary Figure S5: Modulation of apoptosis induction by integrin silencing or pharmacological inhibition in LN308 cells. (**A**) LN308 cells were transfected with integrin β3-, αV-, α4- or ns-siRNA (50 nM) and 48 h later exposed to 100 µM TMZ. After 96, 120 and 144 h the cells were collected by trypsinization, incubated by RNAse and stained with PI for subG1 flow cytometric analysis. The data are the mean of two independent experiments in duplicates ± SD. (**B**) LN308 cells were pre-treated with a cyclo-RGD integrin antagonist (10 µg/mL) alone, or exposed, in addition, to 100 µM TMZ. 120 h later, the cells were subjected to subG1 flow cytometric analysis. The data are the mean of two independent experiments in duplicates ± SD.

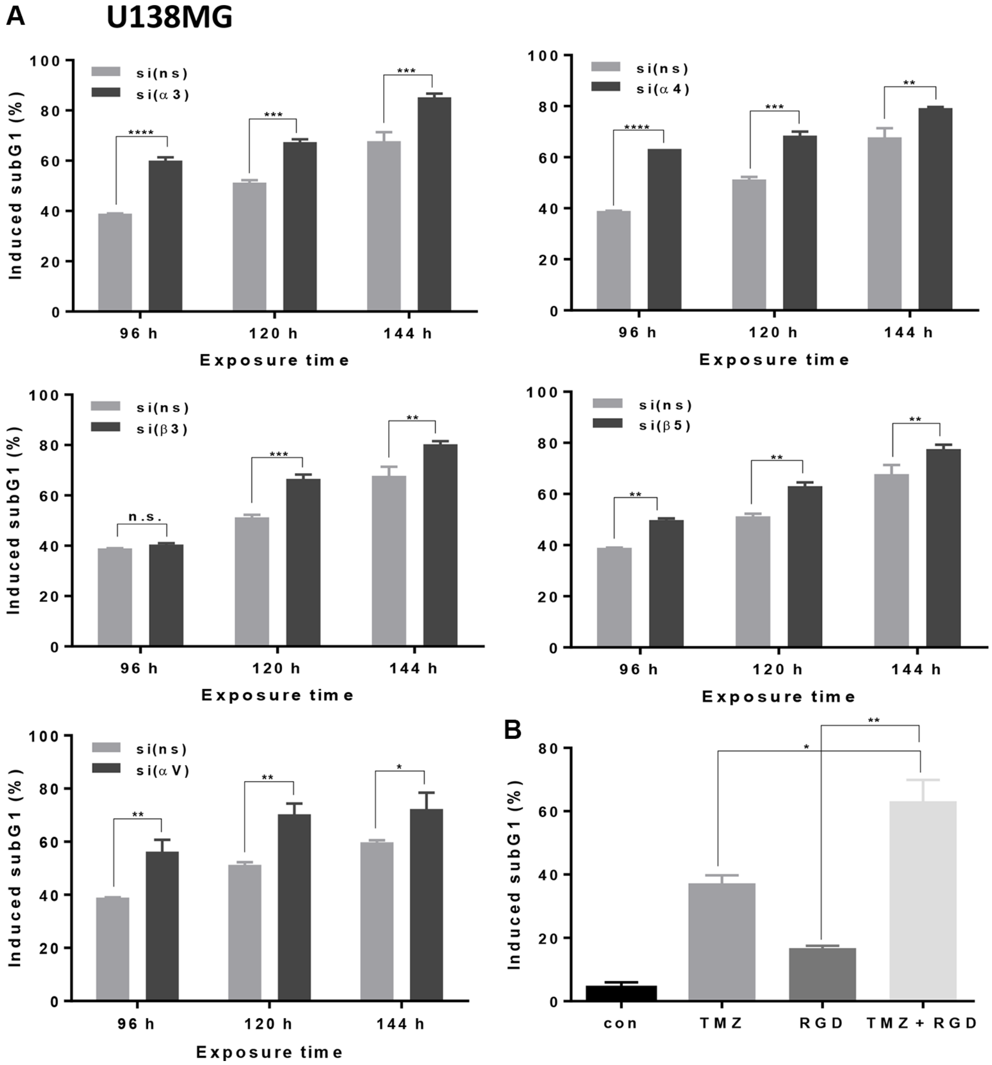

Supplementary Figure S6: Modulation of apoptosis induction by integrin silencing or pharmacological inhibition in U138MG cells. (**A**) U138MG cells were transfected with integrin α3-, α4-, β3-, β5-, αV- or ns-siRNA (50 nM) and 48 h later exposed to 100 µM TMZ. After 96, 120 and 144 h the cells were collected by trypsinization, incubated by RNAse and stained with PI for subG1 flow cytometric analysis. The data are the mean of two independent experiments in duplicates ± SD. (**B**) U138MG cells were pre-treated with a cyclo-RGD integrin antagonist (10 µg/mL) alone, or exposed, in addition, to 100 µM TMZ. 120 h later, the cells were subjected to subG1 flow cytometric analysis. The data are the mean of two independent experiments in duplicates ± SD.

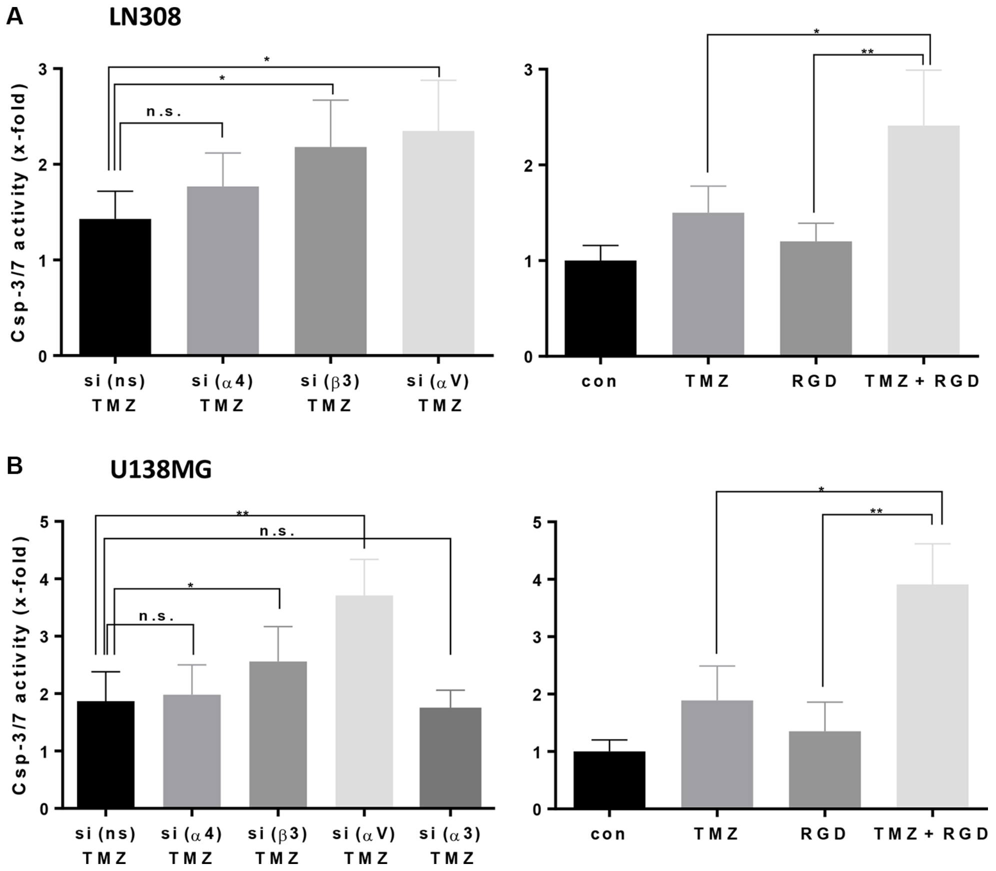

Supplementary Figure S7: Induction of caspase-3/7 activity by integrin silencing or pharmacological inhibition. (**A**, **B**.) LN308 and U138MG cells were transfected with integrin α3-, α4-, β3-, β5-, αV- or ns-siRNA (50 nM) and 24 h later re-seeded in 96-well plates. After additional 24 h cells were exposed to 100 µM TMZ. After 120 h appropriate substrate was added to the cells. Alternatively cells seeded in the 96-well plates were left unexposed or for 120 h exposed the the cyclo-RGD inhibitor (10 µg/mL), 100 µM TMZ or both. One hour after addition of caspase-3/7 peptide substrate, fluorometric signal was measured and the values of the integrin-specific siRNA transfected and TMZ-exposed cells were expressed as x-fold induction relative to the integrin-specific siRNA transfected cells, that were set to 1. The data are the mean of two independent experiments in duplicates ± SD.

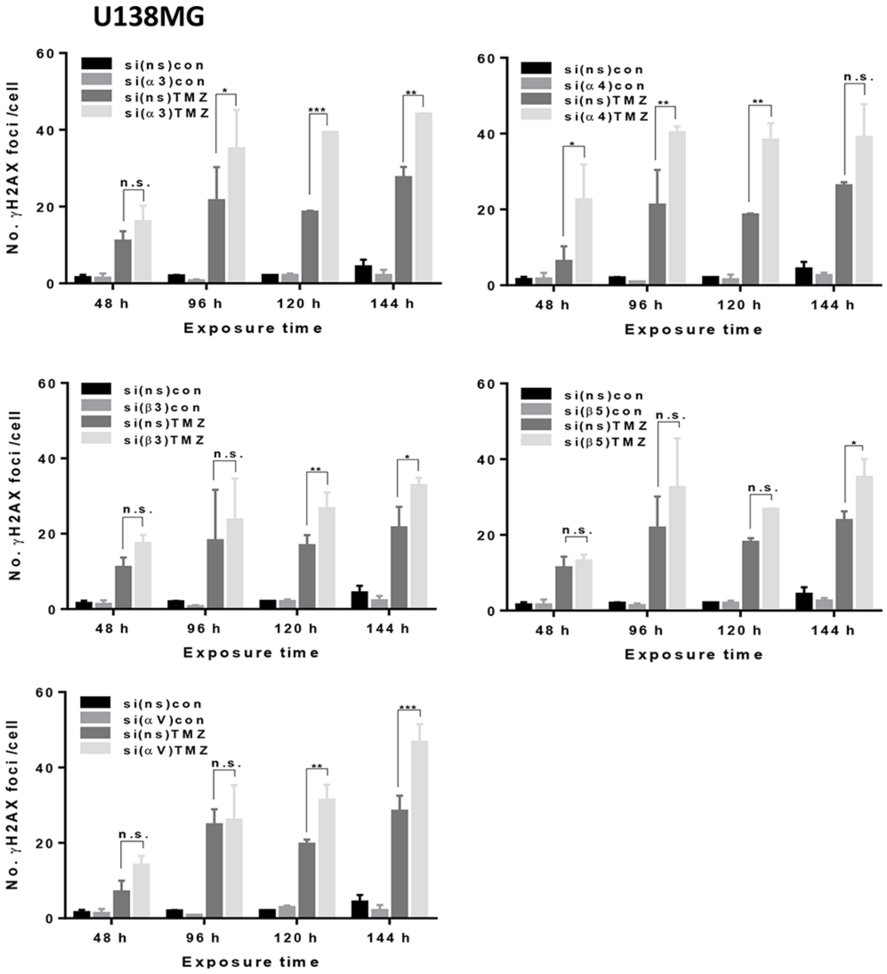

Supplementary Figure S8: Induction of γH2AX foci upon integrin silencing and TMZ treatment. U138MG cells were transfected with integrin α3-, α4-, β3-, β5-, αV- or ns-siRNA (50 nM) and exposed to 100 µM TMZ. After 48–144 h, the cells were fixed on cover slips and stained with anti-γH2AX antibody. Foci (in green) were automatically counted with Metafer4 software. The data are the mean of two independent experiments in duplicates ± SD.

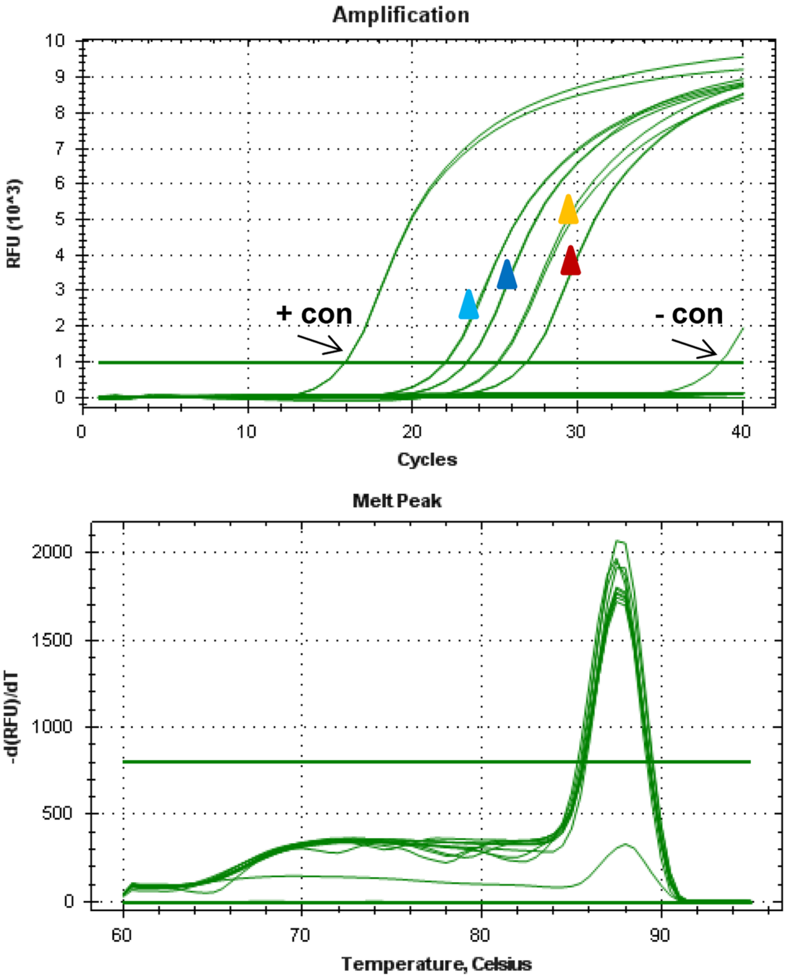

Supplementary Figure S9: HR efficiency assay. Melting curves of the qPCR samples calculated for homologous recombination (HR) efficiency (Norgen Biotek Corp., Canada). Cq for amplification of the positive control plasmid (+con) ~16, for the negative control (-con) showing unrepaired plasmid ~38. HR efficiency differs between the samples, showing Cq values ~22 for ns-si (light blue triangle), ~23 for β3-si (dark blue triangle), ~25 for ns-si/TMZ & TMZ (orange triangle) and ~27 for β3-si/TMZ (red triangle).

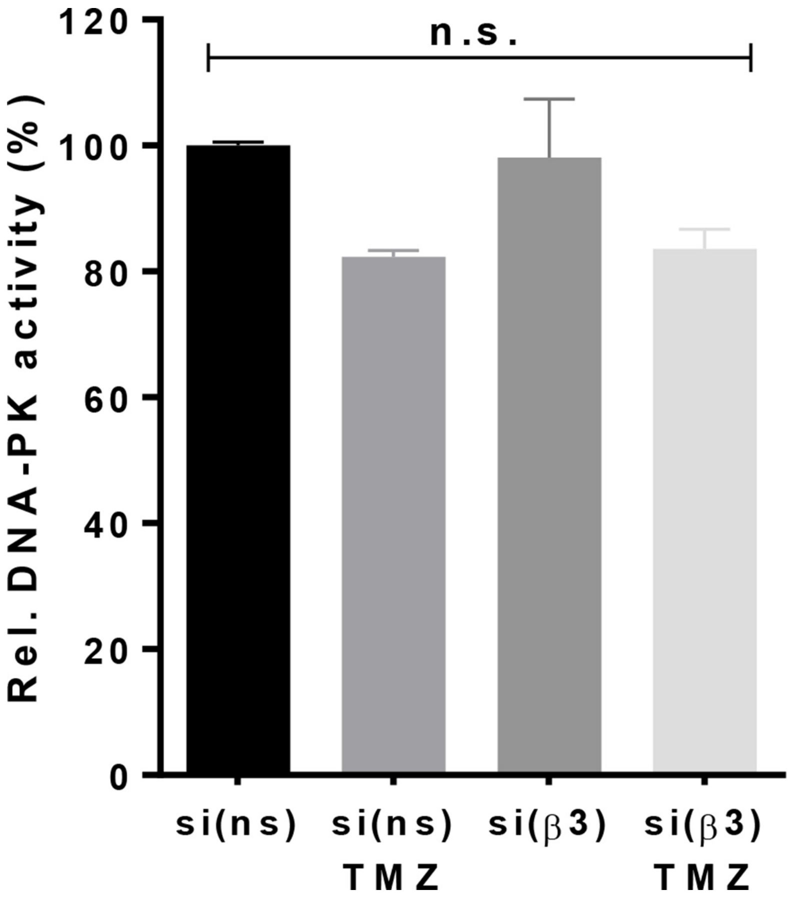

Supplementary Figure S10: : DNA-PK assay. LN229 cells were transfected with si(ns) or β3-siRNA and 24 h later exposed to 100 µM TMZ. DNA-PKcs activity using radioactive assay was performed in whole-cell extracts according to the manufacturer‘s protocol (Promega). n.s., not significant

Supplementary Table S1: Antibody list

Epitope	Antibody type / Source / Dilution
FAK^{y397}	pAb/Abcam/1:2000
FAK	pAb/Abcam/1:2000
Src^{y418}	pAb/Abcam/1:2000
Src	pAb/Abcam/1:2000
Rad51	pAb/Abcam/1:10000
$\gamma H2AX^{S139}$	mAb/Merck Millipore/1:1000 (IF)<br>mAb/Abcam/1:1000 (WB)
H2AX	pAb/Merck Millipore/1:1500
Caspase-3 (p17/19)	pAb/Cell Signaling Technology/1:1000
Pro-caspase-2	mAb/Cell Signaling Technology/1:1000
RIP1	mAb/BD Transduction Laboratories/1:1000
Akt^{S473}	pAb/Cell Signaling Technology/1:1000
Akt	pAb/Cell Signaling Technology/1:1000
IκBα	pAb/Cell Signaling Technology
XIAP	mAb/BD Pharmingen/1:1000
Survivin	mAb/R&D Systems/1:1000
Bcl-xL	mAb/ Santa Cruz Technology/1:1000
β-Actin	mAb/Santa Cruz Biotechnology/1:1000
Hsp90	mAb/Santa Cruz Biotechnology/1:1500
HRP-conjugated anti-mouse/rabbit	IgG/Rockland/1:2000
αVβ3	mAb/Merck Millipore/1:1000
αVβ5	mAb/Merck Millipore/1:1000
α3β1	mAb/Merck Millipore/1:1000
α4	mAb/Merck Millipore/1:1000
goat Alexa Fluor® 488 anti-mouse	mAb/Life Technologies/1:1000
